# Harnessing Insects as Novel Food Ingredients: Nutritional, Functional, and Processing Perspectives

**DOI:** 10.3390/insects16080783

**Published:** 2025-07-30

**Authors:** Hugo M. Lisboa, Rogério Andrade, Janaina Lima, Leonardo Batista, Maria Eduarda Costa, Ana Sarinho, Matheus Bittencourt Pasquali

**Affiliations:** Laboratory of Food Materials and Structure, Food Engineering Department, Federal University of Campina Grande, Campina Grande 58429-900, Paraiba, Brazilmatheus.augusto@professor.ufcg.edu.br (M.B.P.)

**Keywords:** edible insects, functional food ingredients, techno-functional properties, sustainable protein, insect flour, chitosan, life-cycle assessment

## Abstract

The rising demand for sustainable protein is driving interest in insects as a raw material for advanced food ingredients. This review collates and critically analyses over 300 studies on the conversion of crickets, mealworms, black soldier flies, and other farmed species into powders, protein isolates, oils, and chitosan-rich fibers with targeted techno-functional roles. This survey maps how thermal pre-treatments, blanch–dry–mill routes, enzymatic hydrolysis, and isoelectric solubilization–precipitation preserve or enhance the water- and oil-holding capacity, emulsification, foaming, and gelation, while also mitigating off-flavors, allergenicity, and microbial risks. A meta-analysis shows insect flours can absorb up to 3.2 g of water g^−1^, stabilize oil-in-water emulsions for 14 days at 4 °C, and form gels with 180 kPa strength, outperforming or matching eggs, soy, or whey in specific applications. Case studies demonstrate a successful incorporation at 5–15% into bakery, meat analogs and dairy alternatives without sensory penalties, and chitin-derived chitosan films extend the bread shelf life by three days. Comparative life-cycle data indicate 45–80% lower greenhouse gas emissions and land use than equivalent animal-derived ingredients. Collectively, the evidence positions insect-based ingredients as versatile, safe, and climate-smart tools to enhance food quality and sustainability, while outlining research gaps in allergen mitigation, consumer acceptance, and regulatory harmonization.

## Simple Summary

Feeding a growing world while protecting the planet calls for new sources of nutritious, versatile food ingredients. This study explains how insects, such as crickets, mealworms, and black soldier flies, can be carefully processed into powders, oils, and natural fibers that do much more than just add protein. By choosing gentle heat, drying, and grinding steps, we preserve key properties that help bread stay moist, make sauces creamy without the need for added chemicals, and enhance the browning and crispness of snacks. Laboratory tests showed insect powders can hold three times their weight in water, stabilize mixtures of oil and water better than eggs, and form firm gels that improve the texture of meat-free patties. When blended at low levels, these ingredients did not alter the taste or color, and they slowed spoilage by blocking harmful microbes and delaying the onset of fat rancidity. Life-cycle calculations suggest their production uses far less land and emits fewer greenhouse gases than cattle or soy. The findings give farmers, food makers, and policy-makers a practical roadmap for turning farmed insects into safe, tasty, and climate-smart ingredients that can enrich diets, reduce waste, and create new rural jobs.

## 1. Introduction

### 1.1. Global Drivers of Entomophagy

The twenty-first-century food system is confronted simultaneously by population growth, climate change, resource depletion, and socio-economic inequality [1]. Within this context, edible insects have emerged as a promising complement—or even an alternative—to conventional animal- and plant-based foods. The global resurgence of an interest in entomophagy is driven by four interconnected clusters of factors: food security imperatives, sustainability advantages, socio-cultural and economic dynamics, and an increasingly supportive policy–industry landscape [2].

The United Nations projects that the world population will surpass 9.7 billion by 2050, intensifying the demand for foods rich in protein and micronutrients that can be produced with limited resources [3]. Edible insects are exceptionally nutrient-dense, providing complete proteins, essential fatty acids, minerals, vitamins, and dietary fiber in proportions that rival, or exceed, those of meat, dairy, and legumes [4]. Beyond their intrinsic nutritional value, insects offer production efficiencies unmatched by terrestrial livestock; they exhibit superior feed conversion ratios, rapid reproduction cycles, and the ability to thrive on low-value organic side streams or agro-industrial by-products [5]. Such attributes can help close protein gaps in regions vulnerable to malnutrition while reducing the pressure on arable land. The COVID-19 pandemic further underscored the fragility of global supply chains. Locally farmed insects, with minimal cold chain requirements and a negligible zoonotic risk, can serve as a resilient, decentralized protein source that buffers communities against future shocks [6].

Insect farming’s environmental footprint is substantially lower than that of conventional livestock. Life-cycle assessments consistently show dramatic reductions in land occupation, water withdrawal, greenhouse gas emissions, and ammonia volatilization per kilogram of edible protein [7]. Because many edible species grow efficiently on food waste and other organic residuals, insect production can be embedded in circular bioeconomy models that valorize side streams and mitigate landfill burdens. Moreover, harnessing the taxonomic diversity of over 2000 recorded edible species provides opportunities to integrate specific insects into regional agro-ecological niches, thereby enhancing biodiversity, diversifying farmer income streams, and alleviating pressure on overexploited croplands.

Entomophagy is far from novel; it remains a culturally ingrained practice across large swaths of Africa, Asia, Latin America, and Oceania, where insects are lauded for their abundance, palatability, and customary significance [8]. Yet Western markets often perceive insect consumption with skepticism, hindered by neophobia, concerns over hygiene, and lingering misconceptions about safety. For instance, the erroneous belief that insects reared on plant-based substrates might harbor bovine spongiform encephalopathy (BSE)—like prions, despite repeated European Food Safety Authority (EFSA) risk assessments showing no evidence of prion replication in insects—or the unfounded assumption that all edible insects carry high Salmonella loads, even though controlled farming under good manufacturing practices (GMPs) typically yields bacterial counts comparable to poultry or pork, persist [9,10]. Consumer research indicates that acceptance is modulated less by ethnicity and more by socio-economic factors such as urbanization, class, gender, and age [11]. Targeted education campaigns, gastronomic innovation, and transparent safety standards are therefore critical to normalizing insect-based foods and bridging cultural divides.

Regulatory frameworks are rapidly evolving to unlock the potential of edible insects. Agencies in Europe, North America, and Asia have begun approving specific species as “novel foods,” while multilateral organizations encourage research funding and public–private partnerships that scale up production [12,13]. Parallel to this, a new generation of insect-farming enterprises is leveraging waste-fed rearing systems, advanced automation, and precision processing to supply both feed and food markets. As sustainability metrics gain prominence in corporate strategy, the investment in such ventures is projected to accelerate [14].

Collectively, these drivers position insects as a multifaceted solution to some of the most pressing nutritional, environmental, and economic challenges of our time. Nevertheless, the path to mainstream adoption hinges on overcoming cultural reservations, harmonizing safety and labeling standards, and demonstrating a consistent product quality. Continued interdisciplinary research—spanning nutrition science, food engineering, consumer behavior, and policy analysis—will be pivotal in realizing the full potential of insects as novel ingredients for a sustainable global food system.

### 1.2. Overview of Key Edible Insect Species Used in Food Systems

Edible insects form a vast, biologically diverse resource base. Approximately 2200 species are documented as food across 128 countries, with the most extraordinary richness recorded in Asia (932 species) and substantial diversity in Mexico, Africa, and Latin America [15]. Africa alone lists roughly 500 edible species, underscoring the continent’s entomological and culinary heterogeneity [16]. Regular entomophagy is reported in more than 100 nations, predominantly in Asia, Africa, and South America, where insects occupy long-standing roles in local diets [17].

The most widespread groups belong to the orders Orthoptera, Coleoptera, Lepidoptera, Hemiptera, and Isoptera [18]. Beyond being rich in polyunsaturated fatty acids (PUFAs) and monounsaturated fatty acids (MUFAs), several edible insect oils exhibit polyunsaturated fatty acids/saturated fatty acids (SFAs) ratios and low atherogenic–thrombogenic indices that compare favorably with poultry or fish oils. It must be emphasized, however, that these profiles are neither uniform across taxa nor fixed: the proportions of linoleic, α-linolenic, and oleic acids can vary by more than 30% when larval diets are supplemented with oilseeds, cereal bran, or fish off-cuts. Consequently, any nutritional claim based on lipid quality should specify the species, life stage, and feed formulation used [19]. In China, selected insects are valued not only as food but also for their purported medicinal properties, thereby cementing their dual nutritional–health appeal [20].

Most edible insects provide 35–70% protein (dry basis)**;** favorable amino acid scores; digestible lipids rich in essential fatty acids; and appreciable levels of iron, zinc, calcium, B-vitamins, and prebiotic chitin fiber [4,7,21]. Their energy density and micronutrient breadth render them potent tools for combating both protein–energy malnutrition and hidden hunger [19]. It should also be emphasized that the nutritional value of edible insects is highly variable: macronutrient and micronutrient concentrations can differ by more than a factor of two between closely related species and fluctuate further with the sex, developmental stage, rearing temperature, photoperiod, substrate composition, and post-harvest handling [22].

**Figure 1:**
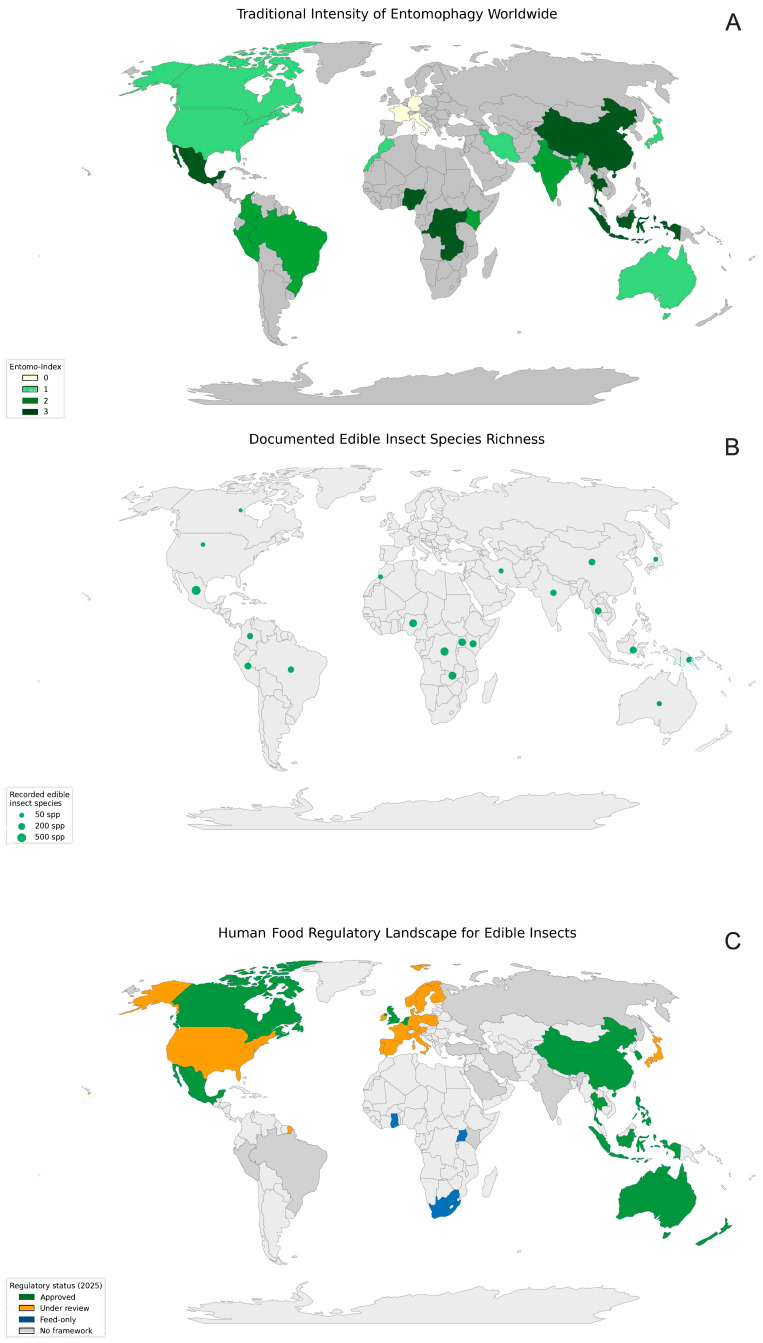
Global entomophagy landscape. (**A**) Cultural reliance on insects (Entomo-Index, 0–3). (**B**) Number of recorded edible insect species (bubble size). (**C**) Food use regulatory status, 2025 (green = approved, orange = under review, blue = feed-only, gray = no framework). Data are drawn from [8,11], Food and Agriculture Organization (FAO) regional reports, and related ethnographic sources.

The combined cultural, biological, and regulatory landscape is summarized in Figure 1, which also illustrates how the regulatory landscape for edible insects is far from uniform.

In regions with a long cultural tradition of entomophagy—such as large parts of Southeast Asia, Central and West Africa, and Latin America—consumption is already covered by broad food safety acts; consequently, these areas appear as having “no insect-specific legislation”. Where insects are a regarded as a *novel* food, for example in the European Union (EU), North America, and Australia/New Zealand, regulators have created dedicated pre-market approval routes—such as the EU Novel Food Regulation 2015/2283, U.S. generally recognized as safe (GRAS) notices, Health Canada’s Food Ingredient Submissions, Food Standards Australia New Zealand (FSANZ) assessments—so these jurisdictions are shown as “under development” or “authorized for selected species”. Economies in which the market is still embryonic (parts of the Middle East and some island nations) have not yet prioritized a bespoke framework, hence the blank or “no data” regions on the map. Overall, the discrepancies reflect four main factors: (i) historical consumption patterns, (ii) consumer acceptance, (iii) the maturity of local insect production industries, and (iv) the capacity and risk assessment philosophy of each food control system.

Depending on the culture and technological development, insects are eaten whole; roasted or fried; ground into flour; or incorporated as protein isolates, oils, or functional ingredients into bakery, pasta, snack, and meat analog products [23]. Their superior feed conversion efficiency, capacity to thrive on agro-industrial side streams, and small spatial footprint position insect farming as a sustainable complement to conventional livestock [24]. However, feed conversion ratios differ markedly among taxa and rearing conditions: crickets (*Acheta domesticus* (Orthoptera: Gryllidae)) raised at 30 °C on protein-balanced diets can achieve dry matter FCRs as low as ~1.3 kg feed kg^−1^ live weight, whereas mealworms (*Tenebrio molitor* (Coleoptera: Tenebrionidae)) and black soldier fly larvae (*Hermetia illucens* (Diptera: Stratiomyidae)) typically require 1.6–2.4 kg kg^−1^, and values > 3 kg kg^−1^ are recorded when temperatures fall or the protein in the substrate is limiting [25,26,27]. Moreover, commercial rearing and processing are increasingly recognized as livelihood engines for rural communities, providing income, employment, and entrepreneurial opportunities [10,28].

Scaling edible insect supply chains requires the strict management of food safety hazards (allergens, microbial pathogens, heavy metals, or pesticide residues) and clear, harmonized regulations, which are still nascent in many jurisdictions [18]. Consumer acceptance remains the principal bottleneck in Western markets, where neophobia and cultural taboos persist [29]. Transparent labeling, culinary innovation, and education are pivotal to overcoming these barriers.

Crickets, mealworms, caterpillars, and other edible insects constitute a nutritionally dense, environmentally efficient, and culturally embedded food resource with a global reach. While Asia, Africa, and Latin America lead in traditional consumption, interest is rising worldwide as sustainability imperatives sharpen. Realizing the full contribution of insects to future food systems will depend on mitigating safety risks, normalizing consumption through gastronomy and marketing, and finalizing robust regulatory pathways.

### 1.3. The Regulatory Status of Edible Insects in Europe and the United States

The physiological discussion in the preceding sections extends beyond the subset of insects currently authorized for human consumption in major markets. A non-exhaustive list of insect species authorized for human consumption in the European Union and/or the United States—together with species frequently studied in the literature but not yet approved—is provided in Table 1. In this table we separated (i) species that have obtained Union-wide authorization as novel foods under Regulation (EU) 2015/2283, (ii) species for which applications are pending or positive scientific opinions exist but no implementing regulation has yet been adopted, and (iii) species that are so far unregulated as food in the European Union and are mentioned here only because they are nutritionally or technologically instructive. For the United States no positive list exists; the Food and Drug Administration (FDA) treats insects explicitly raised for human consumption as conventional foods that must comply with the Federal Food, Drug and Cosmetic Act and Current Good Manufacturing Practice (21 CFR Part 117). Commercial sales in the United States, therefore, concentrate on the same four taxa already cleared in the EU, whereas species such as *Oecophylla smaragdina* (Hymenoptera: Formicidae) or *Lethocerus indicus* (Hemiptera: Belostomatidae) remain confined to ethnogastronomic or research contexts.

This manuscript, therefore, intentionally covers both authorized and as-yet-unregulated taxa because comparative nutritional physiology is valuable for anticipating future authorizations and for guiding breeding or processing research. Nevertheless, any formulation or commercial claim in the European Union must currently be limited to the four species already on the Union list, and a similar caution applies in the United States until a producer has either obtained an FDA “no-questions” GRAS letter or can otherwise document safety under critical good manufacturing practices (cGMPs).

### 1.4. Veterinary Oversight of Insect Farms 

Although farmed insects are invertebrates and therefore fall outside the scope of Directives 98/58/EC and 2010/63/EU that set detailed welfare rules for vertebrate livestock and laboratory animals, several horizontal EU acts—most notably Regulation (EU) 2016/429 [31] on transmissible animal diseases (“Animal Health Law”), Regulation (EU) 2019/4 [32] on medicated feed, and Regulation (EU) 2023/5 [33] on *A. domesticus* as a novel food—already impose hygiene, biosecurity, and record-keeping obligations on insect establishments [34]. These requirements, together with emerging private standards (e.g., IPIFF Guide v2, 2022, [35]), imply that colonies must be monitored for health, density stress, microbiological hazards, and environmental parameters such as temperature, humidity, and substrate quality. We therefore echo recent position papers from the European Federation of Animal Science and the World Organization for Animal Health that call for the creation of dedicated curricula within veterinary medicine, animal science, and production-technology programs covering entomopathology, mass-rearing biosecurity, and welfare assessment tools for *T. molitor*, *A. domesticus*, *A. diaperinus*, and *H. illucens* production systems. Formalizing veterinary competence in the insect sector would professionalize husbandry practices, reduce disease-related losses, improve the end-product quality, and ultimately strengthen consumer confidence in insect-derived foods and feeds.

## 2. Nutritional Composition and Ingredient Forms of Edible Insects

**Figure 2:**
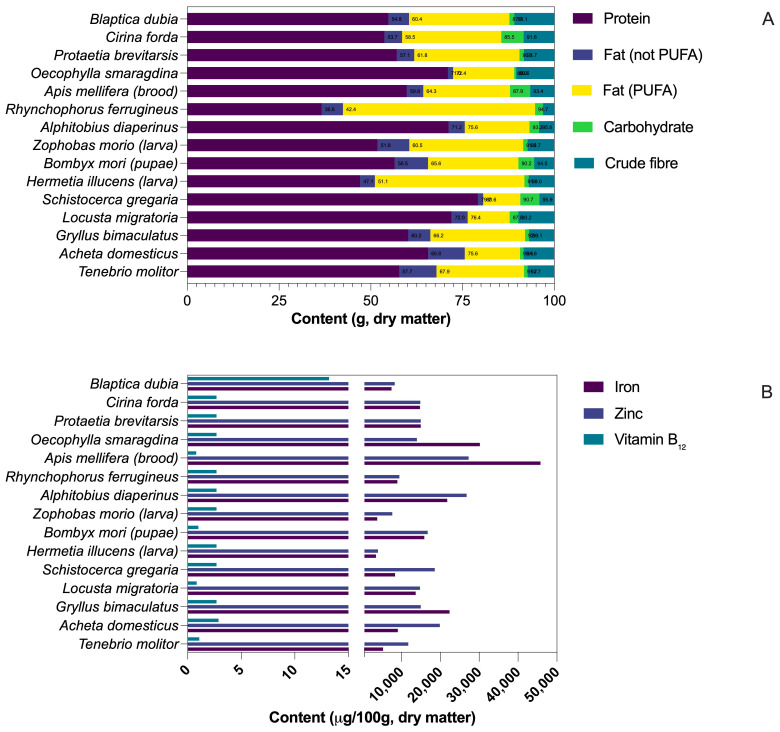
Macronutrient partitioning (**A**) and key micronutrients (**B**) of fifteen widely studied edible insects (dry matter basis). Together with the data reviewed below, the figure illustrates how insects combine a dense supply of protein and lipids with appreciable fiber and micronutrients, all of which can be channeled into distinct ingredient streams through relatively simple processing.

The panels in Figure 2 comprise fifteen taxa that together span the five insect orders most frequently investigated for food and feed—Coleoptera, Lepidoptera, Diptera, Hymenoptera, and Orthoptera—and cover all four species already authorized for human consumption in the European Union as well as several high-volume candidates under review; they therefore provide a representative cross-section of nutrient profiles currently relevant to research and commercialization.

### 2.1. Protein Content, Quality, and Derived Ingredients

The protein content of edible insects spans roughly 20–80% of the dry matter, a range determined chiefly by the taxonomy, developmental stage, and feeding regime. Commercial powders produced from *T. molitor* and the orthopteran *G. assimilis* routinely contain 57–68% protein (dry weight basis). In contrast, composite flours blended from several *Orthoptera* species together with selected *Lepidoptera* species generally provide only 46–53% protein [36]. Adjusting the feed composition can elevate these values further: mealworm larvae raised on pea- and rice-protein-enriched substrates currently hold the record for the highest recoveries reported [37]. Qualitatively, the indispensable amino acid spectrum of most taxa mirrors that found in meat, fish, and poultry. Species such as *I. ertli* provide ample lysine and thereby complement lysine-poor cereal staples [38]. Nevertheless, specific indispensable amino acids may still fall short in a species- and stage-dependent fashion. Typical threshold values at which an the amino acid becomes first-limiting are ≲5% of total amino acids for lysine, ≲1% for tryptophan, and ≲6% for leucine [39]. For example, defatted *T. molitor* larvae contain ≈ 5.5–6.0 g lysine, 1.1–1.3 g tryptophan, and 6.5–7.0 g leucine per 100 g of protein (5.5–6.0%, 1.1–1.3%, and 6.5–7.0% of total amino acids). In contrast, the same species’ pupae drop to ≈4.6% lysine and 5.9% leucine. In *A. domesticus*, tryptophan declines from ≈1.2% in late nymphs to 0.8–0.9% in adults, while adult *Bombyx mori* (Lepidoptera: Bombycidae) pupae may exhibit leucine at only ~5.8% of the total amino acids [22,40]. These examples illustrate how ontogeny (larva → pupa → adult) can markedly alter the indispensable amino acid density, underscoring the need for life-stage-specific nutrient tables when formulating insect-based foods or feeds. Several insects, notably silkworm pupae, meet or exceed FAO/WHO amino acid reference scores [41], and in vitro or in vivo digestibility values of 84–93% place insect proteins among the highest quality animal sources.

Table 2 summarizes some examples of the protein functionality, nutritional quality, and the Protein Digestibility Corrected Amino Acid Score (PDCAAS).

### 2.2. Lipid Fractions and Extracted Oils

Insect lipids rival those of conventional animal foods in quantity yet display strikingly heterogeneous fatty acid profiles. Lipid fraction contents are provided on a dry basis. Larvae of *T. molitor* and *Z. morio* contain 28–40% total fat, of which roughly 30–45% is saturated fatty acids (SFAs), 32–47% is monounsaturated fatty acids (MUFAs; principally 29–35% oleic acid), and 22–23% are polyunsaturated fatty acids (PUFAs; largely 12–15% linoleic acid) [49] (Martins da Silva et al., 2024). Ultrasound-assisted extraction shifts this distribution to about 25% SFAs, 49% MUFAs, and 26% PUFAs [50], while mild steam- or oil-frying likewise elevates PUFAs from ≈23% to ≈29% and lowers SFAs by ~6 percentage points [51]. Taxonomic, sexual, and ontogenetic effects exceed those of processing. In weaver ants (*O. smaragdina*) MUFAs dominate at 51.6% of the total fatty acids (with oleic acid alone at 44.8%), whereas PUFAs contribute only 8.2% and SFAs the remaining 40.2% [52]. Subterranean termites (*Odontotermes* sp.) show an opposite pattern—52.9% SFAs, 44.5% MUFAs, and just 2.6% PUFAs. Sex dimorphism is marked in the giant water bug (*Lethocerus indicus*), and males contain 34.1% SFAs, 42.7% MUFAs, and 23.2% PUFAs, whereas females contain 55.3% SFAs, 31.4% MUFAs, and 13.3% PUFAs [53].

The developmental stage also matters; in the Huhu beetle (*P. reticularis*), pupae attain 38.4% crude fat with a favorable PUFA/SFA ratio of 0.81, compared with larvae at 25.9% fat and a ratio of 0.55 [54]. Crucially, cardiovascular indices remain encouraging after common culinary treatments: both mealworms and Huhu beetles retain atherogenic and thrombogenic indices below 0.3 and 0.2, respectively [54,55]. Specific aquatic taxa reach still higher nutritional values—dragonfly nymphs, for instance, contain up to 6.4% eicosapentaenoic acid (EPA) and 2.1% docosahexaenoic acid (DHA), levels seldom seen in terrestrial insects [56]. The fractionation of these lipid streams offers sustainable alternatives to conventional fats: mealworm oil is enriched in linoleic (37–45%) and oleic (28–38%) acids, whereas black soldier fly oil provides up to 49% lauric acid with recognized antimicrobial activity; the concomitant defatted meal is a protein-dense (≥65% protein, ≤5% fat), shelf-stable co-product [57].

Targeting a specific sex or developmental instar can indeed fine-tune the fatty acid profile, but large-scale implementation remains challenging. In high-throughput rearing facilities, keeping pedigree records or sorting individuals by their sex or instar still relies on manual grading or expensive machine vision systems; both add labor and capital costs that may outweigh the gain in fat quality, so most industrial producers harvest mixed-sex cohorts at a single, late-larval stage and focus on post-harvest refining instead [58,59].

Beyond genetic and ontogenetic factors, the oxidative degradation of insect lipids is a critical quality bottleneck that has received little attention to date. Susceptibility varies markedly among species. Black soldier fly (BSF) fat, dominated by saturated medium-chain lauric acid, shows a naturally high oxidative stability and long induction times [60,61]. By contrast, yellow mealworm oil—rich in polyunsaturated C18 fatty acids—records peroxide values up to 16 meq O_2_ kg^−1^ during storage. They can exceed the 4 mg KOH g^−1^ acid value limit unless antioxidants or low-oxygen packaging are applied [62,63].

Thermal pre-treatments that increase tocopherol levels (e.g., light roasting) or the addition of synthetic or natural antioxidants can delay peroxidation. Meanwhile, nitrogen or vacuum packing and storage below −20 °C further extend the shelf life [62]. Consequently, the fat quality optimization at scale is likely to rely more on inexpensive oxidative stability controls than on costly sex segregation or instar-specific harvesting.

### 2.3. Carbohydrates and Chitinous Fiber

Digestible carbohydrates seldom exceed 10% of the dry matter in edible insects [64]. The dominant polysaccharide is chitin, an insoluble β-(1→4)-linked N-acetyl-D-glucosamine polymer that forms the exoskeleton and contributes between 2.7 and 49.8 g/kg of fresh weight, depending on the species and anatomical fraction [65,66]. Locust exoskeletons contain roughly 12% chitin, surpassing crustacean shells in some cases [67]. Partial deacetylation produces chitosan, a bioactive fiber with antioxidant and antimicrobial properties that broadens functional applications [68]. During protein isolation, chitin accumulates in the insoluble fraction and can be purified further. Insect-derived chitosan compares favorably with crustacean analogs as an edible coating, stabilizer, or soluble fiber additive. At the same time, whole-insect flours naturally deliver chitin within the matrix, thereby enhancing dietary fiber intake [69]. Although chitin may impede mineral bioaccessibility, its prebiotic potential and functional benefits frequently outweigh such limitations when the formulation and processing are optimized.

### 2.4. Micronutrients, Pigments, and Bioactive Compounds

Beyond macronutrients, edible insects are notable for their micronutrient density. Numerous taxa equal or surpass poultry or beef in B-complex vitamins, including riboflavin, pyridoxine, cobalamin, pantothenic acid, and folate, while also supplying fat-soluble vitamins A, D, E, and K and, in some cases, vitamin C [70]. Mineral concentrations of calcium, iron, zinc, phosphorus, magnesium, copper, manganese, and selenium likewise compare favorably with conventional meats, although they vary with the species, life stage, rearing substrate, and processing method [19,71,72].

Insects additionally furnish a suite of specialized metabolites that can be valorized as ingredients. Cochineal (*Dactylopius coccus* (Hemiptera: Dactylopiidae)) remains the commercial source of the red pigment carmine, a colorant valued for its stability in yogurts, confectionery, and beverages [73,74]. Research is expanding to pigments from black soldier flies and silkworms, as well as melanin-like compounds from certain beetle larvae [75]. Larval proteases, lipases, and amylases have been exploited to accelerate fermentation and to tenderize food proteins, demonstrating the potential of insects as biochemical factories [76]. Antimicrobial peptides isolated from insect hemolymph are under investigation as natural preservatives.

### 2.5. Safety and Quality Considerations

While their nutritional promise is clear, edible insects require robust safety management. Some species can bio-accumulate heavy metals, pesticides, or mycotoxins from contaminated substrates, and chitin shares epitopes with crustacean allergens, raising the possibility of cross-reactivity [77,78]. Thermal treatments, defatting, and protein extraction generally reduce the microbial load and antinutritional factors but may also degrade heat-labile vitamins or alter mineral solubility. Consequently, good agricultural and manufacturing practices, substrate control, hazard analyses, transparent labeling, and tailored processing protocols are essential [79]. Bioavailability studies, particularly in human subjects, remain sparse and warrant further inquiry to quantify the absorption efficiency of vitamins, minerals, and chitinous fibers from insect matrices.

Edible insects thus represent a uniquely concentrated and versatile source of high-quality protein, favorable lipids, dietary fiber, and micronutrients. Through relatively low-energy processing, these nutrients can be channeled into whole flours, protein isolates, peptide hydrolysates, extracted oils, chitin-derived fibers, natural pigments, and enzyme preparations, each offering a distinct technological and functional profile. Species selection, life stage targeting, diet optimization, and gentle processing afford tangible levers for enhancing compositions while minimizing safety risks. In summary, insects offer a promising, resource-efficient alternative to conventional livestock products, with the potential to deliver dense nutrition and diverse ingredient functionalities at a fraction of the environmental cost.

## 3. Processing Technologies to Convert Edible Insects into Food Ingredients

Transforming whole insects or fractions of insects into stable, non-recognizable ingredients begins with a three-step unit operation sequence—blanching, drying, and milling—each of which sets boundaries for the safety, shelf life, techno-functionality, and consumer acceptance [80].

### 3.1. Raw Material Preparation

Blanching is typically the first intervention. A brief thermal treatment (usually 1–3 min at 90–100 °C) rapidly inactivates endogenous enzymes, reduces microbial counts, and facilitates subsequent handling, thereby meeting basic food safety requirements [80]. For example, freshly collected grasshoppers or crickets are often placed in boiling water for a minute; this reduces the microbial load and substantially improves hygiene. After blanching, insects may be seasoned and roasted or fried. Roasting is a simple way to develop pleasant flavors and crisp up the insects for direct consumption. Many edible insects are traditionally eaten roasted, toasted, or deep-fried, which not only enhances their taste but also reduces their moisture content, thereby preserving them. Roasting and smoking are also used as primitive drying methods—for instance, some communities smoke dry caterpillars or locusts over a fire, which both cooks and desiccates them for longer storage, [76]. However, as with other animal proteins, heat exposure can reduce thermolabile vitamins and partially denature surface proteins, with knock-on effects on downstream functionality and color [66]. Optimizing time–temperature combinations is therefore essential to balance safety against nutrient retention, and modeling can be regarded as a helpful tool [81].

Although freeze-drying offers a maximal retention of heat-sensitive vitamins and volatiles, its high energy demand limits its industrial adoption. Comparative studies show that well-controlled hot-air- or oven-drying can yield powders of comparable microbiological and functional quality at lower capital and operating costs [82]. Regardless of the technique, achieving a residual moisture content below 5–8% is critical for both microbial stability and efficient milling.

Drying is arguably the most essential traditional method of preservation. By removing moisture, it ensures insects will not spoil quickly and also lightens them for transport. Drying follows immediately to arrest spoilage and create a brittle matrix suitable for the size reduction [83]. Several technologies are in commercial- or pilot-scale use, including solar or microwave-assisted drying and freeze-drying. Methods include sun-drying, meaning spreading insects under the sun, cabinet air-drying, hot-air tunnel or fluidized-bed drying, smoke drying, and, more recently, freeze-drying and microwave drying in industrial settings [84,85]. Drying insects has been shown to increase their microbiological safety, reduce the risk of rancidity, and improve the textural properties of the final product [86]. For example, sun-dried or oven-dried crickets become brittle and can be milled into a stable flour with low water activity. One study highlighted that drying insects to a water activity below 0.6 effectively stops microbial growth and greatly extends the shelf life [87]. Advanced Fick-type models coupling drying kinetics with quality endpoints, recently validated for hydrocolloid-rich grape powders, can be adapted to predict optimum time–temperature paths for insect larvae [83]. Traditionally, insects are often dried after blanching—the blanch step kills most microbes, and the subsequent drying prevents regrowth by creating an inhospitable, low-moisture environment.

In rural settings, sun or smoke drying is a standard method. For example, smoking African caterpillars imparts a smoky flavor, allowing them to be stored for months. In modern processing, freeze-drying is used for premium insect products because it preserves more nutrients and vitamins by sublimating water at a low temperature [88]. Freeze-dried insects maintain their color and heat-sensitive compounds better than hot-air-dried ones [89]. However, freeze-drying is costly and energy-intensive; alternatives, such as microwave drying or fluidized-bed drying, have been explored as faster and more cost-effective methods that still yield a high-quality product. Research shows that while freeze-drying can sometimes cause a higher oxidation of fats, due to the extremely low moisture content, microwave drying achieves a similar low a_w_ with less oxidative damage in some cases [90]. Each drying method can subtly affect the insect quality; for instance, high heat drying might darken insects via Maillard reactions, whereas freeze-dried insects remain light in color but may oxidize more easily. Comparative studies show that well-controlled hot-air- or oven-drying can yield powders of a comparable microbiological and functional quality at lower capital and operating costs [82]. Regardless of technique, achieving a residual moisture content below 5–8% is critical for both microbial stability and efficient milling. Industrially, a combination of steps may be used, blanch → oven-dry or blanch → freeze-dry, depending on the desired product quality and cost considerations.

Milling converts the dried material into flours or fine powders that integrate seamlessly into composite foods and mask the insect’s morphology [91]. Hammer, pin, and cryogenic mills are all employed; particle size distributions below 250 µm improve the mouthfeel, dispersion, and color uniformity while exposing hydrophilic and hydrophobic sites that drive water/oil binding and emulsification. At the same time, lipid oxidation can accelerate if endogenous fat is not removed or stabilized, prompting some processors to incorporate a defatting step before or after grinding [92].

The conventional sequence is increasingly complemented by innovative clean-label technologies. High hydrostatic pressure, pulsed electric fields, ultrasound, and cold atmospheric plasma provide non-thermal routes to inactivate enzymes and microorganisms and for protein unfolding and enhanced lipid/protein extraction [82]. Supercritical CO_2_ and ultrasound-assisted extraction, for instance, improve the fat removal and bioactive recovery while minimizing solvent residues [93]. The industrial scale-up now emphasizes a sanitary design, continuous automation, and the choice between dry or wet fractionation to separate protein, fat, and chitin streams [92].

Processing choices have a significant impact on functional and nutritional quality: mild drying preserves digestibility and antioxidant peptides. At the same time, more intense conditions can increase solubility but diminish gel strength or foaming ability [94]. Moreover, energy use, water demand and global-warming potential vary sharply with technology, influencing the overall sustainability profile [95,96]. From a market standpoint, conversion into fine powders or protein isolates alleviates consumer neophobia and eases incorporation into familiar foods.

In sum, blanching, drying, and milling establish the critical foundation for safe, shelf-stable, and functionally versatile insect ingredients. The precise control of these operations—augmented by emerging non-thermal and green extraction techniques—enables processors to tailor both the techno-functionality and environmental footprint, thus facilitating the seamless integration of insects into mainstream food systems.

**Figure 3:**
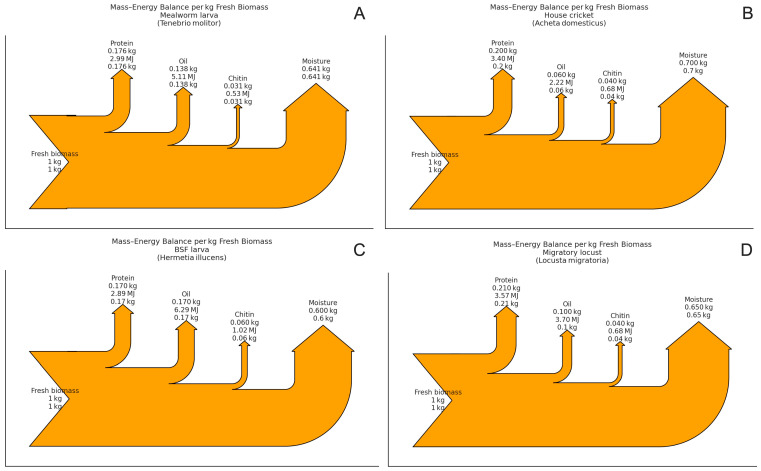
Comparative mass and chemical energy balances (Sankey diagrams) for 1 kg of fresh biomass from four commercially relevant edible insects processed through the blanch → dry → mill chain. Panels show the partitioning into protein, oil, chitin and moisture streams—with the intrinsic chemical energy (MJ) annotated on the solid fractions—for the (**A**) mealworm larva (*T. molitor*) [97], (**B**) house cricket (*A. domesticus*) [98], (**C**) black soldier fly larva (*H. illucens*) [99], and (**D**) migratory locust (*L. migratoria*) [100]. The consistent layout highlights the higher lipid yield of larval species (**A**,**C**) and the leaner protein-rich profile of adult Orthoptera (**B**,**D**).

Figure 3 compares the mass and energy flow profiles of four widely farmed species processed through the blanch–dry–mill chain, illustrating how larval insects yield proportionally more oil, while adult Orthoptera are comparatively protein-rich.

Across all four species, 60–70% of the incoming mass is lost as evaporated water during drying—thus the prominent moisture arrow. What sets the insects apart is the composition of the remaining ~30–40% solids. Larval beetles such as *T. molitor* and dipteran larvae *H. illucens* allocate a significantly larger portion of their dry weight to lipids (≈14–17% wet basis), while adult/juvenile Orthoptera (crickets and locusts) dedicate that biomass to myofibrillar protein. As a result, lipids represent over 50% of the chemical–energy flow in the two larval species, but only ~25% in crickets and ~35% in locusts, where protein becomes the main energy carrier.

For oil-rich larvae, defatting is crucial: recovering ≥ 0.14 kg oil kg^−1^ of fresh biomass unlocks a high-value energy-dense stream (≈5 MJ), leaves a low-fat protein cake with improved functionality, and reduces oxidative rancidity during storage. In contrast, cricket and locust processing should prioritize maximizing the protein solubility and gelling, as their leaner (~6–10% fat) matrices already resemble conventional meat in terms of protein-to-energy ratios. Chitin yields are similar (0.03–0.06 kg kg^−1^) across all species, providing a consistent but secondary stream for the fiber or chitosan extraction. Overall, the diagram highlights that the same unit operation train yields quite different value propositions, such as an oil and protein biorefinery for larvae versus a high-yield protein isolate for adult Orthoptera—insights that can inform both equipment selection (e.g., oil presses vs. salt-assisted protein extraction) and techno-economic models of an integrated insect plant.

### 3.2. Protein Extraction and Fractionation

The efficient recovery of insect protein hinges on an integrated extraction–fractionation chain that begins with judicious pre-treatments and ends with ingredient tailoring to specific functional targets. Wet fractionation remains the benchmark for high-purity products: insects are blanched, defatted, and dispersed in water, and the soluble proteins are precipitated at their isoelectric point (pH ≈ 4.5–5.0) [101]. Coupling isoelectric precipitation with ultrafiltration routinely delivers concentrates exceeding 80% protein, although the overall recovery can be limited (~28%) because chitin-bound and heat-insoluble fractions are lost [102]. In contrast, dry fractionation—fine milling followed by air classification or sieving—eliminates the use of water and chemicals. While yields are lower and the protein purity tops out around 55–60%, the drastically reduced energy, water, and solvent footprint makes dry methods attractive for low-moisture applications. Efficacy is strongly influenced by the prior blanching, freeze-drying, and solvent or supercritical CO_2_ defatting, which loosen the exoskeleton and increase particle friability [103,104].

To bridge the purity–yield gap, processors employ salt-assisted extraction. Moderate ionic strengths (0.3–0.5 M NaCl) “salt-in” myofibrillar-like insect proteins, enhancing solubility. After this, dilution or dialysis “salts-out” the proteins as highly functional, with >70% pure isolates. These salt-derived proteins exhibit superior emulsifying activity, water- and oil-holding capacities, and heat-induced gel strength compared to whey or soy benchmarks [105]. Enzymatic hydrolysis offers a complementary route: the controlled protease treatment (e.g., alcalase or trypsin) of defatted insect meal breaks down macromolecules, releasing peptides with a greater solubility across a wide pH range and improved foaming and antioxidant properties [106]. When hydrolysis is followed by the membrane cut-off concentration, peptide-rich fractions with tailored molecular weight distributions can be produced for beverages, sports nutrition, or flavor applications.

Pre-treatments influence downstream success. Defatting increases protein concentrations and eliminates lipid-borne off-flavors; solvent extraction optimizes lipid removal but risks denaturing proteins, while supercritical dioxide carbon (SC-CO_2_) balances the yield with the native functionality [107]. The choice of drying method impacts the color, particle fragility, and subsequent solubility. Freeze-dried, defatted powders typically provide the highest extraction yields and the lightest color. Innovations such as low-shear microfiltration enable the simultaneous reduction in the bioburden and fractionation, producing protein streams that are both foaming- and gelling-competent, which can surpass conventional dairy proteins in model systems [108].

Functionally, insect protein ingredients obtained through these routes demonstrate water/oil-binding, emulsifying, foaming, and gelation capacities that are equal to or superior than those of soy and whey isolates [94]. Amino acid profiles remain balanced, with essential amino acid contents rivaling soy and surpassing most cereals; protein-enriched dry fractions routinely reach 55–58% protein while wet isolate pathways exceed 80% [105]. Such versatility permits incorporations into baked goods, meat analogs, shakes, feed premixes, and dietary supplements, where the “invisible” powder form significantly improves consumer acceptance [104].

The central challenge is process optimization: tailoring the pre-treatment severity, pH swings, ionic strengths, enzyme type, and hydrolysis time to maximize the overall yield without compromising techno-functionality [108]. Because each method yields fractions with distinct compositions and rheological behaviors, a toolbox approach—combining dry and wet routes or sequential salt extraction and enzymatic hydrolysis—allows manufacturers to match the ingredient functionality to product needs [106]. In summary, advances in aqueous, dry, salt-assisted, and membrane-based technologies now enable the scalable production of high-quality insect protein ingredients, unlocking their potential as sustainable alternatives in diverse food and feed formulations.

### 3.3. Lipid Extraction and Defatting Methods

Optimizing the lipid removal from insects serves two parallel objectives: (i) generating a valuable fat fraction rich in medium- and long-chain unsaturated fatty acids and (ii) producing low-fat protein meals with superior functional and sensory properties. A variety of extraction strategies—conventional solvents, supercritical fluids, aqueous/mechanical systems, and newer intensification techniques—have thus been benchmarked for their yield, selectivity, oxidative stability, and energy demand.

Conventional solvent extraction remains the industrial reference. Non-polar solvents, such as hexane, chloroform, or methyl-tert-butyl-ether (MTBE), penetrate the hydrophobic cuticle and dissolve neutral lipids efficiently. Chloroform–methanol (2:1) mixtures often produce the highest recoveries from crickets, locusts, and other orthopterans, whereas MTBE excels for silkworm pupae. Across most taxa, hexane and MTBE consistently deliver a high protein enrichment in the defatted cake (≥65% protein) without disturbing the native molecular weight profile [109,110]. Supercritical CO_2_ (SFE), operated at 30–40 MPa and 45–60 °C, can match solvent yields for lipid-rich species such as mealworms but may underperform for leaner house crickets; even so, black soldier fly larvae processed under SFE can yield up to 43% fat—equivalent to hexane—with the added benefit of sequentially recovering carotenoids, sterols, or vitamin E in later extraction steps [111].

Where solvent use is undesirable, aqueous and mechanical routes emerge as greener options. Aqueous extraction, which involves grinding insects in water followed by centrifugation, typically recovers only 40–60% of the total lipids. However, combining freeze-drying with mechanical screw-pressing increases yields and produces fats with a markedly superior oxidative stability compared to solvent-stripped equivalents [112]. Pre-heating or steam blanching softens the cuticle, enhancing the oil release during pressing.

Process intensification has concentrated on ultrasound-assisted extraction (UAE) and pressurized liquid extraction (PLE). Short bursts of high-power ultrasound in an ethanol–water (60:40) mixture disrupt cellular matrices, selectively enriching polyunsaturated fatty acids (PUFAs) while simultaneously reducing cholesterol [50]. Similarly, PLE at 10 MPa using hot ethanol enhances the ω-3: ω-6 ratio and shifts the lipid class distribution toward nutritionally favorable phospholipids. A “one-pot” high-speed homogenization method employing a binary ethanol/hexane solvent on wet biomass has also proven to be scalable; the biphasic solvent outperformed single solvents in both lipid recovery and protein retention, reducing the energy input by half compared to dry-milling routes [113].

The choice of extraction directly affects the protein quality in the residual meal. Hexane or MTBE defatting increases the crude protein content by 20–25% and enhances indispensable amino acids without altering electrophoretic profiles; solubility peaks at an alkaline pH, particularly when lipids are removed with MTBE or hexane [104]. Conversely, aggressive ethanol or chlorinated solvents can partially denature proteins, thereby reducing their solubility. Lipid-side process variables modulate the fatty acid composition: UAE and PLE enrich PUFA proportions, improve atherogenic/thrombogenic indices, and increase oxidative stability indices compared with untreated oils [114].

Oxidative stability is governed by cross-interactions between slaughtering, drying, and defatting. Freezing insects before processing minimizes lipase activity and peroxide formation, whereas freeze-drying followed by mechanical pressing yields the most stable fats during 12 weeks of storage at 25 °C. By contrast, blanching coupled with SFE accelerates the formation of secondary oxidation products, indicating that moist-heat pre-treatments can open reaction sites that are later exploited by supercritical CO_2_ [112]. The measured antioxidant capacity of the final oil closely mirrors these trends, underscoring the importance of the sequence design.

From a sustainability perspective, the energy demand clusters around water removal, with dewatering and thermal drying accounting for 40–80% of the total process energy in dry-milling/solvent routes. Microwave-assisted drying and real-time moisture sensing can help reduce this footprint, but further optimization, such as integrating low-pressure vapor compression or solar drying, is necessary for large-scale viability [113,115].

In summary, solvent, supercritical, mechanical, and assisted extraction platforms each offer distinct trade-offs in terms of the yield, composition, stability, and environmental impact. By tailoring the solvent polarity, pressure–temperature schedules, and pre-treatment severity, processors can fine-tune both the nutritional value of insect-derived lipids and the functional quality of the accompanying protein ingredients, cementing insects’ role as a versatile, sustainable raw material for next-generation food and feed.

### 3.4. Emerging Non-Thermal and Advanced Thermal Technologies

A new generation of mild processing tools are being evaluated to dry, stabilize, or fractionate insects while maximizing nutrient retention and functionality. These approaches either avoid bulk heating altogether or apply heat so rapidly and uniformly that the chemical and sensory degradation is minimized.

Microwave and infrared (IR) heating deliver volumetric or surface-directed energy at far higher power densities than conventional ovens [116]. In practice, house crickets, mealworms, and grasshoppers reach an a_w_ ≤ 0.3 within minutes, yielding shelf-stable powders at energy inputs up to 40% lower than those of hot-air-drying and at costs far below those of freeze-drying [117]. Rapid moisture removal suppresses Maillard browning and protects heat-labile B vitamins and polyunsaturated fatty acids; IR roasting simultaneously develops desirable nutty notes with minimal surface scorching. Similarly to probiotic acerola juice, where hydrocolloids and mild spray-drying preserved >90% cell viability, insect-derived peptide powders benefit from pairing gentle dehydration with tailored gum matrices [118]. The main engineering challenge is the scale-up—a uniform energy distribution must be maintained in larger, heterogeneous insect beds to avoid local overheating.

High-pressure processing (HPP) subjects the packaged insect purée, pâté, or high-moisture extrudate to 400–600 MPa for a few minutes at <40 °C [119]. The isostatic pressure inactivates vegetative microbes and most enzymes without perceptible cooking, preserving the color and delicate aromas while extending the chilled shelf life two- to three-fold. Pressure also weakens the chitin–protein matrix, yielding smoother textures in spreads or beverages and facilitating downstream wet-milling. Because the spore inactivation is limited, HPP products still require cold chain distributions or the use of hurdle combinations.

Ultrasound-assisted extraction (UAE) applies high-intensity (20–40 kHz) waves to insect slurries, creating cavitation that ruptures cuticular structures and liberates intracellular proteins, lipids, and phenolics. Compared with static alkaline extraction, UAE can raise the protein yield by 15–25% and shorten the extraction time from hours to < 30 min, while simultaneously producing smaller protein aggregates that show a superior solubility, emulsifying activity, and gel strength [120,121]. Short sonication bursts can be coupled with defatting or isoelectric precipitation, resulting in negligible increases in energy demands.

Ohmic heating passes an alternating current directly through insect slurries, generating heat uniformly through internal resistance and reaching target temperatures in seconds. This rapid, electrode-mediated heating effectively inactivates native proteases and lipoxygenase, limits flavor deterioration, and can serve as a pre-treatment before fermentation or dehydration. Preliminary studies report an improved color and a 25% reduction in lipid oxidation markers compared to conventional blanching; however, electrode fouling and conductivity control in high-fat matrices remain areas for optimization [122].

Finally, enzymatic hydrolysis with food-grade proteases (e.g., alcalase, trypsin, flavourzyme) converts defatted meals into peptide hydrolysates with a >90% in vitro digestibility. Controlled hydrolysis (degree of hydrolysis (DH) ≈ 10–20%) enhances the solubility across pH 3–8, boosts the foaming capacity, and imparts savory umami notes suitable for broths and seasonings. Notably, breaking down high-molecular-weight allergens, such as tropomyosin and arginine kinase, can attenuate IgE reactivity, providing a pathway to hypoallergenic insect ingredients for both human foods and high-value pet diets.

Collectively, these non-thermal or advanced thermal techniques provide processors with modular options to tailor insect ingredients, combining rapid drying or pasteurization with intensified extraction or allergen management, while maintaining nutritional integrity and reducing energy footprints relative to legacy operations.

## 4. Functional Properties and Roles of Insect-Derived Ingredients in Foods

### 4.1. Functional Properties

A summary of the functional properties of selected insects is presented in Table 3.

#### 4.1.1. Water- and Oil-Holding Capacity

The ability of insect-derived ingredients to bind water lipids is a crucial functional trait for modulating the texture, retaining moisture, and stabilizing fat in complex food matrices. Protein isolates obtained from *T. molitor* larvae retain up to 3.95 g of water per g of dry matter. At the same time, defatted flours from the saturniid caterpillar *Arsenura armida* absorb 275.6% of their own weight in water, values that are equal or exceed those of many conventional plant proteins [123]. The lipid affinity is similarly high: *G. sigillatus* protein preparations adsorb 3.33 g of oil per g, and the same *A. armida* flour captures 121% of oil on a weight basis [128].

The species, structural form, and processing history strongly modulate the functional performance. The removal of endogenous fat concentrates protein and exposes additional hydrophilic and hydrophobic sites, resulting in consistent improvements in both the water-holding capacity (WHC) and oil-holding capacity (OHC) [129,130]. Non-thermal technologies, such as high hydrostatic pressure, pulsed electric fields, and related methods, can further enhance these properties by partially unfolding proteins and increasing the surface area, thereby facilitating water and lipid binding [131]. Across several comparative studies, purified protein preparations outperform whole or partially defatted flours, underscoring the influence of the particle size distribution and matrix composition on functionality.

In practical applications, a high WHC mitigates cooking losses and improves juiciness in meat analogs. In contrast, an elevated OHC stabilizes fat-rich emulsions and extends the shelf life in baked goods and snack products [132]. Because the insect lipid composition can be manipulated through dietary interventions and protein functionality can be optimized by gentle processing, formulators possess considerable latitude to tailor the ingredient performance for specific product architectures [126,133].

#### 4.1.2. Emulsification and Foaming Behavior

The interfacial performance of insect proteins rivals—and, in some cases, surpasses—that of conventional plant and dairy emulsifiers. Enzymatic hydrolysis, sonication, and defatting all disrupt quaternary structures, expose hydrophobic residues, and lower molecular weights, thereby improving the diffusion to oil–water or air–water interfaces. Hydrolyzed locust protein, for example, displays an emulsifying activity index 54% higher than its native counterpart at pH 7 [103], while sweep frequency ultrasound yields grasshopper extracts with an emulsion stability of 85.5% [94]. Protein isolates from *G. sigillatus* achieve a 72.6% emulsifying activity—which is substantially above values reported for whole or even defatted flours [129]—underlining the advantage of protein purification. Beyond wet extraction, spray-dried protein–polyphenol micro-particles exhibit > 60% emulsion stability and afford improved oxidative protection, hinting at multifunctional roles in high-lipid formulations [134,135].

The foaming capacity follows similar trends. Defatting augments the surface hydrophobicity and can raise the foam expansion by roughly 30% relative to full-fat flours [123], although excessive lipid removal may compromise long-term stability [136]. Controlled hydrolysis is particularly potent: locust hydrolysates show a 326% increase in foamability at pH 3, attributable to an optimal peptide size and net charge. Ultrasonic treatments further enhance both the expansion and stability across a broad pH spectrum, presumably by generating flexible, partially unfolded peptides that form cohesive interfacial films [137].

Taken together, insect proteins constitute adaptive emulsifiers and foaming agents whose performance can be fine-tuned by mild physicochemical treatments. By modulating the hydrolysis degree, sonication intensity, or lipid removal, formulators can tailor insect-derived ingredients for beverages, bakery aeration, meat analogs, and emulsion-based sauces, expanding the sustainable functionality palette available to the food industry [94,136,138,139].

#### 4.1.3. Gelation, Viscosity, and Texturizing Potential

Insect proteins exhibit an innate ability to form three-dimensional networks, provided that the pH, ionic strength, and thermal input are correctly tuned. Studies on honey-bee brood proteins demonstrate gelation thresholds comparable to those of egg and soy proteins, with an optimum firmness developing at pH 5–7 after heating to 85 °C [132]. Recent work shows that targeted enzymatic cross-linking can further push those gel limits: microbial transglutaminase reduced the critical gelation concentration of *T. molitor* isolates by ≈35% and doubled G′, effectively compensating for the species’ low intrinsic disulfide density [140]. The effect of upstream processing is critical: high hydrostatic pressure or controlled enzymatic hydrolysis can increase the solubility and surface activity. For example, HHP at 400 MPa boosted the house cricket protein solubility by 25%. Still, it simultaneously decreased the water-holding capacity and gel hardness—underscoring that non-thermal preservation tools must be paired with micro-structural diagnostics and not judged on solubility alone [141]. However, these same treatments do not always translate into stronger gels or more stable foams, underscoring the delicate balance between the peptide size, charge distribution, and network formation [142]. The same studies confirm that “more soluble” is not synonymous with a “better gel”; once the degree of hydrolysis exceeds ~10%, peptide chains become too short to support a continuous network, so the solubility rises while the gel firmness and foam stability collapse [143].

The rheology of composite doughs likewise responds sharply to insect inclusions. The incremental replacement of wheat flour with whole insect powders elevates viscosity and promotes pronounced pseudoplastic behavior; at an equivalent loading, *Locusta migratoria* generates the most significant increases in both storage (G′) and loss (G″) moduli, yielding stiffer, more elastic matrices suitable for texturized snacks or pasta [144]. Such viscoelastic reinforcement arises not only from protein–starch interactions but also from the considerable water-binding and oil-binding capacities already documented for many insect preparations [145]. Crucially, rheological reinforcement does not automatically translate into superior baking: the loaf volume drops by 12–18% once mealworm powder exceeds 15% of the flour, and panelists the rate crumb dryness unacceptable above a 10% inclusion level, whereas desert locust flour retains acceptable sensory scores up to ~10% [146,147]. Emerging high-moisture extrusion data further show that soy matrices can tolerate up to ~30% insect protein isolate before the tensile strength plateaus; beyond that, the isolate particle size (<50 µm) rather than the inclusion level dictates the fiber integrity in the final meat analog [148].

Textural engineering can be further refined through gentle fractionation. Defatting concentrates protein and increases the hydrophilic surface area, often enhancing gel strength. At the same time, spray-drying creates low-density powders whose particle morphology improves the dispersion and network continuity in batters and emulsified meat analogs [149]. In baked systems, the addition of *A. diaperinus* flour increases the breaking force and crispness, illustrating how species-specific proteins and fibers modulate crust formation and water migration during thermal processing [144].

These functionalities are still being optimized; the research is in a relatively early, “exploratory” stage for insect proteins [150]. Factors such as the insect species, protein extraction method, degree of defatting, and processing aids, including the pH adjustment and enzyme treatment, significantly influence performance. Interestingly, using insect *protein isolates*, as opposed to whole insect meal, tends to enhance functional properties across the board [151]. This is because the isolation removes other components that might dilute or interfere with the protein functionality.

In conclusion, from a functional standpoint, insect-derived ingredients have considerable promise as multifunctional food additives. With continued research into processing techniques (e.g., pH-shifting, ultrasonication, and enzymatic hydrolysis), it is likely that the functional performance of insect proteins can be tailored to meet specific food formulation needs, potentially positioning insect proteins as alternatives to egg, soy, or dairy proteins in various products.

#### 4.1.4. Antioxidants and Antimicrobial

Beyond their nutritive value, insect-derived ingredients can also contribute to food preservation through their inherent antimicrobial and antioxidant compounds. Table 4 provides a summary of the bioactive functional properties of selected insect ingredients.

A prominent example is chitosan, derived from insect chitin [68]. Chitosan is known to have a broad-spectrum antimicrobial activity and is used as a natural preservative and edible film in foods [161]. Insect-sourced chitosan, present in the shells of crickets or cockroaches, has been shown to inhibit bacteria such as *E. coli* and *S. marcescens* and to scavenge free radicals when formed into a thin film. Chen et al. (2021) found that a chitosan film derived from the American cockroach (*Periplaneta americana* (Blattodea: Blattidae)) had a ~72% DPPH radical scavenging activity and significantly suppressed bacterial growth, suggesting it could serve as an active food packaging material [162]. Thus, insect chitosan can function as a natural preservative coating, prolonging the shelf life of perishables by reducing microbial spoilage and oxidative degradation. Similarly, incorporating chitosan or chitin-rich insect fiber into food can impart some antioxidant stability and antimicrobial effects internally, although high levels may affect the texture, as demonstrated elsewhere [65].

Insect proteins and peptides also demonstrate antioxidant properties. When insect proteins are hydrolyzed, they often release peptides that can donate electrons or chelate pro-oxidant metals, acting as antioxidants [163]. Research has identified specific antioxidant peptides in silkworm pupae, crickets, and other insects that exhibit vigorous radical-scavenging activity [164]. For example, peptides like SWFVTPF (from mealworm) have been isolated with a high antioxidant potency. Although these peptides are typically studied for health benefits, they could also help stabilize foods by preventing the oxidation of fats. An insect protein hydrolysate added to a meat product or oil may slow rancidity due to the presence of peptide antioxidants. Some insect species also contain phenolic compounds or carotenoids derived from their plant-based diets, which are present in insect extracts and contribute to their antioxidant capacity.

Another preservation angle is the use of antimicrobial peptides (AMPs) produced by insects [165]. Insects rely on potent AMPs as part of their immune system. For instance, defensins and cecropins from insects can kill bacteria and fungi [166]. While extracting these compounds at scale from insects is not yet practical for food use, there is potential to utilize insect farming by-products as a source of natural antimicrobials to protect food. More simply, the lauric acid-rich fat of black soldier fly larvae is known to have antimicrobial effects against specific pathogens [167]. If insect oils are used in a formulation, they may contribute some antimicrobial action, although it is likely to be mild. Finally, fermented insect products (like the mealworm paste) often develop organic acids and bacteriocins from fermentation microbes, which can lower the pH and inhibit spoilage organisms, similarly to how fermented fish sauce resists spoilage [168]. Thus, if used as an ingredient, a fermented insect extract could carry preservative organic acids or mild bacteriostatic compounds.

In summary, insect-derived ingredients can play a functional role in food preservation, including chitosan for antimicrobial films, antioxidant peptides to delay oxidation, and possibly other bioactive compounds to maintain food quality. This aligns well with the clean label trend of using natural preservatives instead of synthetic additives [169,170]. As research progresses, we may see a more targeted use of insect-sourced preservative systems—for instance, as a sachet containing insect chitosan in a bread package to inhibit mold growth or an insect peptide blend added to high-fat foods to extend their shelf life. These applications make insect ingredients not only nutritious but also technologically functional in enhancing food stability and safety.

### 4.2. Functional Roles

#### 4.2.1. Flavor and Aroma

The flavor chemistry of edible insects is remarkably heterogeneous. Headspace analyses of more than 40 species have identified over 250 volatiles, whose relative abundance—and hence sensory impact—shifts with the taxonomy, rearing substrate, and life stage. Key contributors include lipid-derived aldehydes and ketones (hexanal, 2-nonenal), sulfur compounds such as methional, and Maillard pyrazines that impart roasted or peanut-like notes, particularly in *T. molitor* and *A. domesticus* powders. This biochemical diversity explains why sensory panels describe some species as pleasantly nutty and cereal-like, while others evoke “earthy”, “green”, or even marine aromas unfamiliar to many Western consumers [171,172,173]. 

Because flavor unfamiliarity is a principal driver of food neophobia, most ingredient-processing schemes therefore start by dampening the characteristic “earthy/animal” off-note, for example, through defatting, solvent-washing, pH-shift extraction, or controlled thermal/enzymatic treatments, all of which have been shown to improve the sensory liking and purchase intent [174,175]. This is additionally supported by the findings from the defatting of yellow mealworm flour (ethanol Soxhlet), which removed odor/flavor negatives and restored the consumer liking of cereal bars to control levels [176]. Similarly, the solvent defatting (using hexane, ethanol, and acetone) of the *G. bimaculatus* protein concentrate improves techno-functional traits and reduces volatile off-compounds [130].

Immediate blanching or steaming after harvest inactivates lipoxygenase and other enzymes that liberate grassy aldehydes. Rapid, low-moisture drying, such as microwave, infrared, or freeze-drying, then removes volatile precursors while limiting secondary oxidation. Several studies show that blanch-plus-microwave protocols decrease ammonia-like compounds by more than half relative to slow oven-drying, while maintaining their color and microbiological safety [64,82]. Defatting is equally decisive: the hexane or supercritical-CO_2_ extraction of cricket or mealworm meal eliminates most unsaturated lipids that oxidize into rancid aromas, yielding flours that sensory panels rate up to 25% higher in overall liking [176,177]. Beyond suppression, processing can generate desirable savory notes. Controlled proteolysis (Alcalase, papain) and mixed-culture fermentation release glutamate, short peptides, and 5′-nucleotides, creating an intrinsic umami boost. A fermented *T. molitor* seasoning sauce, for instance, accumulated >7 g/L of free amino acids and was judged to be comparable to soy or Worcestershire sauce on the on the umami intensity, demonstrating insects’ potential as novel savory bases [178,179]. 

Thermal roasting provides a complementary route by intensifying Maillard chemistry, as dried insects roasted at 140–180 °C yield Strecker aldehydes and alkyl pyrazines that rise sharply, delivering toasted, cocoa-like nuances that translate into pleasant, nutty notes in downstream flours. For example, Bawa et al. (2020) substituted wheat flour with 5% and 10% house cricket (*A.domesticus*) powder in pan bread; sensory panels (n = 30) detected no change in the overall liking at the 5% inclusion and only a modest decline at 10%, while the protein content rose from 8.9 g to 12.7 g (100 g^−1^) [180]. Similarly, Ruszkowska et al. (2022) fortified corn–rice extrudates with 10% cricket flour; 105 consumers rated the overall acceptability as 7.1 ± 1.2 on a nine-point hedonic scale, which is significantly higher than the 6.4 ± 1.3 scored by the insect-free control [181]. 

Concerning nutrient retention, Żołnierczyk and Szumny (2021) reported that roasting *T. molitor* larvae at 160 °C for 15 min elevated total pyrazines eight-fold yet reduced the extractable protein by only ~5% and left in vitro digestibility above 85% [182]. Taken together, these studies show that the judicious use of roasted insect flours can enhance the toasted–nutty flavor complexity and consumer acceptance while essentially preserving their protein enrichment value.

Moderate inclusion levels (≤10% *w*/*w*) of such flours in bread or extruded snacks consistently enhance the flavor complexity without dominating the matrix, and consumer trials in Europe and Asia report an acceptance equal to or higher than that of the control product [183,184]. Should residual “buggy” volatiles remain at higher fortification rates, formulators resort to spice blends, smoke flavors, or microencapsulation to mask them, strategies shown to restore liking scores to baseline in cereal bars and pasta [82,185]. Hydrocolloid-stabilized nano-emulsions, such as the guar/xanthan systems that retained thyme oil volatiles for controlled release, offer a direct template for trapping “grassy” aldehydes from insect lipids [139].

Despite these advances, several research gaps persist. Quantitative libraries linking specific volatiles to processing parameters are still fragmentary, which limits the predictive control of the flavor during scale-up. Systematic kinetic studies on enzyme inactivation, drying rate effects, and lipid oxidation are needed, as a more profound exploration of tailored fermentations could craft signature flavor profiles (e.g., nutty–cocoa vs. meaty–broth). Finally, psychophysical work on detection thresholds for insect-specific volatiles would inform maximum inclusion levels across product categories. Addressing these gaps will enable processors to pivot between creating virtually flavor-neutral insect isolates for “stealth” fortification and harnessing insects’ innate umami potential for next-generation natural seasonings [172]. 

#### 4.2.2. Texture and Structure

When whole insects are milled, defatted, or fractionated, the resulting powders and isolates behave much like conventional functional proteins, principally by binding water and oil, thickening batters, and stabilizing emulsions. Comparative techno-functional screens show that protein extracts from *T. molitor*, *A. domesticus,* and *L. migratoria* display a significantly higher WHC and OHC than their parent flours, owing to the removal of lipids and chitin that otherwise crowd active binding sites [150]. Mealworm isolate, for example, binds up to 3.9 g of water per g of protein, essentially matching soy (≈3.0 g/g) and mung bean proteins [186,187]; incorporated at 7–10% into high-protein bars or yeast breads, it softens the crumb and delays staling by retaining moisture. In bakery matrices, insect flours also act as bulk thickeners: replacing 6–10% of cereal flour with cricket powder slightly darkens the crumb but produces muffins and pasta of acceptable chewiness when water is adjusted [188,189]. In gluten-free doughs, the viscoelastic deficit can be partly offset by 2–6% defatted cricket powder, which improves the gas retention and loaf volume while reducing the crumb firmness after storage [190,191,192].

Insect proteins additionally gel during heating. The rheological work indicates that many isolates gel at a neutral-to-alkaline pH with strengths comparable to eggs or soy; heating mealworm protein at pH 7–8 yields cohesive networks suitable for sausages or tofu analogs [193,194]. In emulsified meat batters, substituting 15–20% of pork with an insect isolate reduces the cook loss and increases the bite firmness without a sensory penalty, confirming its role as a binder–emulsifier [194]. For a more complex structure, high-moisture extrusion can align insect proteins into fibrous strands that mimic whole-muscle meat. Partially defatted mealworm or cricket flour extruded at 140–160 °C under ≥60% moisture forms anisotropic lamellae with a chewiness comparable to soy-based meat analogs [148,195]. Although such extrudates still lack some juiciness, blending insect protein with plant isolates or adding clean-label gums is narrowing the textural gap with chicken breast.

Structural contributions also come from insect polysaccharides, including chitin and its deacetylated derivative, chitosan. Insect-derived chitosan forms weak gels and edible films under mildly acidic conditions and is being trialed as a protective coating on cheeses and fresh fruit, where it reduces moisture loss and microbial spoilage while adding a slight “bite” to the surface [196,197]. Insoluble chitin particles left in minimally refined flours can provide dietary fiber and impart a subtle crispness or viscosity in high-fiber cookies and snack pellets.

Taken together, insect ingredients can thicken, bind water and fat, gel, emulsify, and build fibrous matrices, with a performance tunable through defatting, pH-shifting, enzymatic pre-digestion, or extrusion. Mastering these textural levers is essential for mainstreaming insect-based foods, whether as stealthy fortifiers in bread or as the centerpiece of plate meat analogs.

#### 4.2.3. Appearance and Color

Insect ingredients can influence the appearance of foods in two main ways: by contributing color/pigments or by being essentially invisible to the consumer. In product development with insects, the goal is often to ensure the final appearance is familiar and appetizing, with no visible insect particles or unusual colors that might deter consumers [198]. For the most part, finely milled insect flours or isolates integrate homogeneously. For example, pale cricket flour in a cookie dough might slightly speckle the dough with brown, but no obvious insect pieces remain [199]. Using lighter-colored insects—like the yellow mealworm, which is light after removing its brown head—or removing the dark exoskeletal parts can yield a flour that is not noticeable in color. In protein isolates, most pigments are removed, resulting in an off-white powder that, when added to foods, has a minimal effect on the color [200]. This is advantageous for maintaining the expected appearance of foods (e.g., a protein-enriched pasta still looks like regular pasta).

However, some insect flours do impart color. Darker insects—like black soldier fly larvae, which are black when mature—produce very dark meals that can turn a dough gray-brown—often an undesired effect [201]. A study comparing different insect flours in bread found that cricket and mealworm flours produced a brownish color similar to whole-grain bread, whereas black soldier fly flour made the bread considerably darker and less appealing [202]. One solution is to blend insect flour with other flours to dilute the color or to use *bleaching* steps on the flour, using food-safe bleaching or simply removing the darkest body parts, to improve the color. In extrusion cooking, the high temperature can also darken insect ingredients via Maillard browning, which needs to be controlled [203].

On the other hand, insect-derived colorants can be intentionally used to impart the desired color. The clearest example is carmine, the red color extracted from cochineal insects, as discussed earlier. Carmine and cochineal extract have been used for centuries to impart a vivid red hue to foods [204]. They are valued for their excellent heat and light stability, making them superior natural red dyes in products such as candies, ice cream, juices, and cured meats. While carmine’s insect origin is sometimes viewed as a negative (for vegan or allergy reasons), it demonstrates that insect-derived ingredients can meet stringent color performance requirements in the food industry. Cochineal is essentially an insect transform that is no longer recognizable at all; it is just a soluble dye, yet provides a function (color) that consumers do notice and enjoy [205].

Besides red, other colors from insects are being studied. For example, *silkworm pupae* contain a pigment that can produce a yellow-brown dye, and some grasshoppers have greenish pigments [206,207]. Additionally, the structural color of certain insect shells (iridescence) is not directly applicable to foods, but novel research is looking at using ground beetle shells as a glittery food decoration. Another appearance-related aspect is the surface finish: an insect-derived product called *shellac* (from lac insects) is widely used as a glazing agent on candies and fruits, producing a shiny appearance. Shellac is a resin secreted by insects used to coat apples or confectionery for a glossy finish. This, too, is a way insects indirectly contribute to the appearance of mainstream foods [208].

In summary, processed insect ingredients are typically designed to be *neutral in appearance* when incorporated—they should not alter the color or appearance of the food. Yet insects also offer natural colorants like carmine that are prized in their own right for their vibrant, stable colors. This dual role is interesting: on one front, technologists strive to eliminate any visual trace of insects in products (to ensure consumer acceptance), and on another front, specific insect-derived compounds are added because of the visual appeal they provide. Balancing these considerations is part of the formulation strategy when working with insect-based ingredients.

## 5. Applications in Food Formulation and Product Development

Harnessing the above functional properties, food scientists and product developers have begun incorporating insect ingredients into a variety of food products. The goal is often two-fold: to improve the nutritional profile and to maintain acceptable sensory qualities. Table 5 provides a summary of the applications of insect-sourced ingredients in food products.

### 5.1. Bakery Products (Breads, Biscuits, and Snacks)

One of the most common approaches is blending insect flour into cereal flour for breads, crackers, cookies, or pasta. Even a modest inclusion level of insect powder can significantly increase the protein content. Studies have found that substituting about 5–10% of wheat flour with cricket or mealworm flour in baked products is feasible without adverse effects on the taste or texture [224]. For instance, bread with up to 10% cricket flour and biscuits or cookies with ~5% cricket flour were deemed acceptable to consumers in trials [225]. Such enrichment typically improves the protein content, amino acid balance, and mineral levels of the product. In one case, adding 10% cricket flour to bread raised the protein content noticeably and improved the bread’s amino acid profile (adding lysine, which wheat is low in) [202]. Likewise, biscuits fortified with palm weevil larvae flour—in high amounts, with up to a 70% replacement of wheat flour—showed a protein content increase of ~45%, along with a higher fat and energy content yet still achieved positive sensory scores [211]. Another study on cricket-enriched bread and muffins reported improved nutritional profiles and higher protein and fiber contents, with a generally positive acceptance, especially when the insect content was kept moderate [226]. The key to acceptability is often that the insects are milled into a fine flour and are well incorporated, so the consumer does not detect identifiable insect pieces. With a proper formulation using savory ingredients such as spices and chocolate, it is possible to mask any subtle insect taste. Products like cookies, chips, protein bars, and breakfast cereals containing insect ingredients have been developed and well-received in various markets. Notably, a recent report mentioned cookies enriched with palm weevil larvae were not only more nutritious but also scored high on the sensory and acceptability evaluation [227]. This demonstrates that insect ingredients can be successfully incorporated into familiar forms, delivering nutrition without deterring consumers.

### 5.2. Pasta and Noodles

Insect flour has also been added to pasta dough to increase its protein quality [212]. For example, pasta made with ~10–15% cricket or grasshopper flour has a higher protein and iron content and is generally comparable in taste to regular pasta [198,228], with perhaps a slightly darker color. Research on mealworm-fortified pasta revealed an improved essential amino acid content and only minor changes in texture when cooked, which consumers found acceptable in blind tastings, especially when served with sauce [229]. These prototype products indicate that entomophagy can be introduced subtly via everyday staples.

### 5.3. Meat Products and Extenders

Insects can be used in meat formulations or even as meat replacers [230,231]. Because insect proteins can gel and bind water, they are suitable as extenders in comminuted meat products, such as sausages, patties, and meatballs [232]. For instance, supplementing ground meat with 5–10% insect powder, such as cricket or locust powder, has been tested [233]. It boosts protein and mineral contents, reduces the overall fat percentage if the insect flour is defatted, and has only a minor impact on flavor. Some studies have created hybrid beef burgers with 10% mealworm paste, finding that the patties remained juicy and scored well in consumer tests for taste, with the main difference being a slightly darker color [234]. Additionally, insect flour can serve as an emulsifier in emulsified sausages, potentially reducing the need for other binders [235].

Taking a more innovative leap, insects are being used to create entirely new foods that mimic the taste and texture of meat. High-moisture extrusion (a process to produce fibrous meat-like structures from plant proteins) has been applied with insect ingredients. In one study, cricket flour combined with a soy protein isolate (15–30% cricket content) was extruded to form a meat analog with a fibrous texture [217]. The inclusion of the cricket slightly reduced the strength of the fibers at higher levels. Insects lack the gluten-like network of wheat or specific plant proteins. Still, at 15% and 30% inclusions, they successfully produced a structured “meat” with an anisotropic index of up to 2.8 [217,236]. This suggests that insect proteins can participate in forming meat-like fibers when combined with other proteins under specific conditions. The result was a product akin to a meat substitute that could be flavored and used like chicken strips. Similarly, extrusion has been used to produce insect-enriched snacks such as puffed extruded chips [220]. Adding insect flour to a cereal-based extruded snack formula can increase its protein content by several percent and add nutrients, although extrusion parameters often need adjustments as the insect material can alter the expansion and crunchiness [237]. Nonetheless, extruded insect snacks, such as cornmeal and cricket flour extruded curls, have been produced with an acceptable texture and taste, thereby broadening the range of palatable insect-based foods available.

### 5.4. Dairy Analogs and Beverages

While less common, insect proteins have been utilized in high-protein beverages and dairy-like products [238,239]. Researchers have incorporated insect protein powder into protein shakes, noting that it can be as effective as whey or soy protein in boosting the protein content. However, flavor masking is crucial since insect protein can have a distinct “earthy” or nutty taste [240,241]. There are also experiments using insect flour as an ingredient in fermented products; for instance, one study replaced a portion of milk solids with buffalo worm flour in a yogurt-type product and found that it achieved a similar protein content and fermentation properties [242]. In line with symbiotic Petit Suisse prototypes fortified with inulin and whey [243], a cricket protein yogurt-type product can combine prebiotic fibers with insect peptides for added functionality. The fermented product had higher iron and zinc levels, although the color was slightly off-white. Ensuring an appealing flavor and appearance is the main challenge, but it also demonstrates the potential for insects to be used beyond solid foods, possibly in future fortified drinks or supplements.

## 6. Sustainability and Life-Cycle Considerations

Edible insects have been widely heralded as a more sustainable protein source compared to both conventional livestock and some plant-derived ingredients. A growing body of life-cycle assessment (LCA) studies supports this claim, showing that insect farming can significantly reduce key environmental burdens, such as greenhouse gas (GHG) emissions, land use, and water use relative to traditional animal proteins [10,25]. For example, producing 1 kg of edible protein from mealworm insects was found to generate far fewer greenhouse gas emissions and use only a fraction of the land compared to making the same amount of protein from milk, chicken, pork, or beef [244]. One benchmark study reported that mealworms require only about 10% of the land needed for beef protein production (and ~43% of that for milk), while emitting considerably less GHGs per unit of protein than chickens, pigs, or cattle. Such reductions are highly significant given that conventional livestock currently contributes ~15% of the global anthropogenic GHG emissions and occupies ~70% of agricultural land [245]. Replacing even a portion of meat protein with insect-based ingredients could thus substantially shrink the carbon and land footprint of the food system. A recent scenario analysis in Europe projected that substituting insect biomass for meat could cut GHG emissions associated with meat production by approximately 72–97%, with the most significant benefits seen in replacing beef and the smallest (though still sizable) in replacing poultry [246]. These figures underscore the climate mitigation potential of insects as an alternative protein source.

Feed conversion efficiency is a critical factor underlying these sustainability advantages. Insects are poikilothermic (cold-blooded) and can allocate more of their energy to growth rather than metabolic heat. As a result, they boast very high feed conversion ratios compared to mammals and birds [247]. For instance, crickets need roughly six times less feed than cattle *and* half the feed of broiler chickens to produce an equal amount of protein [10]. This efficiency means that to yield a given quantity of protein, insect farming requires far less feed input and thus indirectly saves on the land, fertilizers, and energy that would otherwise be on expended growing feed crops for livestock [248,249].

Additionally, because insects can be farmed in dense vertical systems, the direct space (or land footprint) required is minimal. Mealworms, for example, can be produced in stackable trays indoors, resulting in land use requirements per kilogram of protein that are orders of magnitude lower than those of grazing or even intensive livestock farming. One LCA found the land footprint of mealworm protein to be only ~10% of beef’s on an equivalent protein basis [25].

The water use for insect cultivation is also significantly lower than for traditional livestock. Insects do not need to drink much water if their feed is moist, and they have high water retention efficiency. A water footprint analysis demonstrated that obtaining protein via mealworm farming is considerably more water-efficient than via cattle or pig farming [248]. Mealworms were reported to require about 23 L of water per gram of protein, whereas beef needed roughly five times more (over 110 L/g protein). In comparative terms, mealworm protein’s water footprint was found to be lower than that of beef or pork and on par with or slightly better than the most water-efficient animal protein (chicken) [250]. These findings suggest that insects could also alleviate pressure on freshwater resources. It is worth noting, however, that results can vary with methodologies—some studies indicate insect farming’s water use can exceed poultry’s in specific scenarios. For example, when insects are reared in climate-controlled facilities with dry feed, there may be hidden water inputs upstream (irrigation for feed crops, etc.) [251]. Nonetheless, across most analyses, insects emerge as comparatively water-thrifty protein producers, especially when accounting for the water needs of feed production.

Beyond these direct comparisons, LCA studies on edible insect production consistently confirm a favorable environmental profile while also highlighting the importance of rearing conditions. In a review of the LCA literature, it was observed that insects generally have lower GHG emissions per kilogram of protein than pork or beef. However, they may be slightly higher than poultry in some cases [252]. Crucially, the choice of the rearing substrate (diet) has a significant influence on the outcome [253]. If insects are fed high-quality grain-based feed, like conventional feed for poultry or pigs, their environmental impacts include the upstream effects of the feed production. In contrast, feeding insects on low-value side streams, such as food waste or agricultural by-products, can dramatically improve the LCA results. Smetana et al. (2016) demonstrated that using food industry by-products (like distillers’ grains) as insect feed yielded some of the best sustainability outcomes, whereas using dedicated high-protein feeds incurred higher impacts despite greater yields [253]. This aligns with other studies showing that insects raised on waste streams can partially offset the burden of producing their feed, sometimes even leading to net positive environmental services by recycling waste. For example, black soldier fly (BSF) larvae can be grown on organic waste, reducing that waste’s mass by bioconversion while producing insect biomass. Bava et al. (2019) found that rearing BSFs on agro-industrial by-products not only yields protein but also significantly reduces the waste volume that would otherwise require disposal [254]. Such integration of insect farming into waste management exemplifies a circular economy approach, where nutrients from waste are looped back into the food chain through the use of insects.

It is essential to emphasize that the scalability and industrialization of insect production are crucial to realizing these sustainability benefits. Current commercial insect farms are relatively small compared to livestock operations, and many LCAs rely on pilot data or lab-scale estimates. As production scales up, there are opportunities to further enhance efficiency through automation, climate control optimization, and genetic improvements, which could drive even lower impacts. Oonincx and de Boer, 2012, noted that mealworm farming was still in its infancy and that productivity gains through better automation, feed optimization, and selective breeding could substantially reduce the impacts per kg [25]. Indeed, historical gains in poultry and swine efficiency (~2–3% productivity improvement per year) suggest insects could undergo similar or greater improvements as farming technology matures. However, scaling up also means that processing and energy considerations take precedence. Insect rearing in temperate climates often requires artificial heating to maintain optimal growth temperatures, which can increase the energy consumption and related emissions if non-renewable energy sources are used [255]. For example, one study found that the energy use in a Dutch mealworm facility resulted in energy expenditures slightly higher than those in broiler chicken production, though still comparable to or lower than those in pork and beef [256]. The source of energy, renewables vs. fossil fuels, will play a significant role in the future GHG profile of large insect farms. Similarly, maintaining ventilation and lighting in indoor farms adds to the electricity demand. Encouragingly, the high density and year-round production potential of insects mean that, if paired with renewable energy and waste heat recovery, the overall energy use per protein can be minimized.

Another key consideration is the post-harvest processing of insects into food ingredients, which itself carries an environmental footprint. Turning whole insects into powders, flours, oils, or protein isolates involves several steps, including blanching, drying (often via freeze- or oven-drying), grinding, oil pressing, or solvent extraction and sometimes protein solubilization and precipitation. Each step consumes energy and water and may involve auxiliary materials such as solvents. These processing stages can contribute non-trivially to the product’s life-cycle impacts [257]. For instance, conventional meat supply chains account for 15–25% of their GHG emissions that arise after the farm gate, which is attributed to slaughtering, chilling, and transportation [258]. Similarly, if insect ingredients undergo substantial processing, their cradle-to-gate footprint will reflect these added burdens.

There is evidence that highly refined insect products may partly erode the sustainability edge. One report noted that producing a defatted insect protein concentrate can demand significant energy for drying and defatting, making its impact closer to, though still lower than, animal protein isolates [259]. Even plant protein isolates are not impact-free: producing soy protein isolate, for example, requires significant inputs of water and energy for extraction, sometimes resulting in a footprint approaching that of certain meats [260]. Therefore, it is crucial to assess the net benefits after processing. If insects are consumed in moderately processed forms, they retain more of the environmental advantage. More exhaustive processing will yield a more functional ingredient, but at the cost of a higher energy use and, possibly, chemical usage. It was noted that, currently, there is no standardized processing method to convert insects into human food; however, any future processing and storage requirements will add to the impacts similarly to how slaughtering and cold chain handling add impacts for conventional meats. Consequently, research into efficient processing technologies will be crucial to maintaining a low overall footprint when producing insect-based ingredients at scale.

In summary, insect-derived food ingredients exhibit a markedly favorable sustainability profile compared to traditional animal ingredients and even some highly processed plant proteins. By virtue of the superior feed conversion and the possibility of rearing on waste streams, insects offer substantial reductions in GHG emissions, land occupation, and water use per unit protein [261]. The circular economy potential is particularly notable—insects can upcycle side streams into valuable nutrients, thereby closing nutrient loops and mitigating waste treatment emissions [262]. LCAs indicate that under optimized scenarios, insect protein can be 2–5 times more environmentally efficient than conventional animal proteins. However, real-world outcomes will depend on scaling responsibly, which involves controlling energy uses, sourcing sustainable feed substrates, and implementing best practices in farming and processing. Ongoing assessments are necessary as the industry evolves, but the current evidence strongly supports insect-based ingredients as a promising component of more sustainable and resilient food systems.

## 7. Future Directions and Research Gaps

Despite the considerable progress in harnessing insects as food ingredients, significant research gaps and technical challenges remain before insect-derived ingredients can fully realize their potential in the food industry. This section highlights key areas for future research, ranging from the nutritional and functional optimization of insect ingredients to improvements in processing technologies, safety assessments, and novel applications such as cultured insect cells. A tight alignment with the central theme—converting whole insects into stable, functional, and non-recognizable food components—is maintained throughout.

### 7.1. Nutritional Quality and Techno-Functional Optimization

Edible insects already provide all the indispensable amino acids and an attractive spectrum of micronutrients, yet their protein quality and functionality remain highly variable across species and processing routes. The main culprit is chitin: when flours are milled from whole larvae or adults, the fibrous polysaccharide forms a physical barrier that restricts protease access, driving the true ileal digestibility and, consequently, the PDCAAS down to 0.44–0.81—far below the ≈0.97 typical of casein or egg protein [263]. Recent meta-analyses therefore recommend replacing the PDCAAS with the more stringent DIAASs, which is more sensitive to the effects of chitin and other antinutritional factors [150]. Systematic studies that couple mechanical de-shelling with mild chitinase treatments are urgently required to decouple protein and chitin without sacrificing the nitrogen accuracy or creating excessive free amino nitrogen, which could promote off-flavors.

The protein quality is further modulated by rearing and post-harvest conditions: the insect diet, drying temperature, defatting intensity, and thermal load can each shift the essential amino acid balance by as much as 15% and alter digestibility by comparable margins [264]. Multifactorial experiments—ideally harmonized around the INFOGEST in vitro digestion protocol and reporting DIAASs—are needed to map these variables across the approximately twenty insect species most likely to reach the industrial scale.

The functional performance is equally immature. The native solubility of many insect proteins is <20% at a neutral pH, limiting their use in high-protein beverages or emulsions; yet targeted alcalase- or flavourzyme-mediated hydrolysis has already increased the solubility of migratory locust and mealworm proteins to ≈55%, with parallel gains in the emulsifying activity and foam stability [103]. High-pressure processing, ultrasound, and pH-shifting are also under exploration, but structure–function data sets linking sequence motifs, such as glycosylation patterns and disulfide density, to interfacial behavior remain scarce. Advanced proteomics, coupled with rheology and interfacial tensiometry, can close this gap and inform process windows that maximize functionality without excessive hydrolysis.

To accelerate translation, three community deliverables are proposed: (i) an open database compiling DIAASs, solubility, and interfacial metrics for the major farmed species under standardized conditions; (ii) consensus specifications for “insect protein isolates” (≥80% protein, DIAASs ≥ 0.90, solubility ≥ 60% at pH 7) analogous for those that exist for soy and dairy; and (iii) safety and life-cycle dossiers quantifying how emerging de-chitinisation and hydrolysis streams influence allergenicity, Maillard reaction products, and heavy metal accumulation. Addressing these intertwined nutritional and functional questions will move the field from exploratory proofs-of-concept toward reproducible, scalable processes that convert whole insects into stable, performance-tailored, and consumer invisible food ingredients.

### 7.2. Flavor and Consumer Palatability

Another pressing hurdle is the sensory optimization of insect ingredients. Converting insects into fine powders or protein isolates removes the visual cue but does not eliminate flavor and aroma issues. Raw or lightly processed flours from mealworms, crickets, and grasshoppers have been described as “earthy,” “nutty,” “shrimp-like,” or “hay-like,” reflecting their distinctive lipid- and amino acid-derived volatile profiles [168,261]. When the inclusion level in breads, snacks, or pastas exceeds roughly 10–15%, these notes become readily perceptible and can curb consumer acceptance [262]. Systematic flavor modulation strategies are therefore needed. Controlled roasting or fermentation has been shown to shift the volatile spectrum of cricket and mealworm preparations towards sweeter, roasted, and caramel-like notes, thereby improving sensory scores [265,266]. The deodorization of crude insect oils can strip sulfurous off-notes and permits higher fat replacement levels in bakery applications, while leaving the fatty acid profile intact [267]. Microencapsulation offers an additional route to mask residual off-flavors and protect unsaturated insect lipids from oxidation during storage and processing [268,269]. Finally, manipulating the rearing diet—analogous to feed–flavor links in conventional livestock—has emerged as a farm-level lever: the substrate composition alters the insect’s fatty acid and volatile profile and, in turn, its sensory quality [270,271]. Despite these promising leads, the flavor chemistry of edible insects remains sparsely mapped; identifying the key odorants and quantifying how each processing step amplifies or suppresses them is an open research frontier that must be addressed to enable a broad consumer acceptance of insect-derived food ingredients.

### 7.3. Safety, Technology, and Market Translation

Allergenicity is the foremost safety uncertainty. Tropomyosin and arginine kinase, dominant crustacean allergens, occur in most farmed insects, and clinically relevant IgE cross-reactivity has been documented, including anaphylaxis after a seasoned cricket snack [272,273,274]. Species- and process-resolved allergen maps, together with trials on how heat, fermentation, or proteolysis modulate epitope potency, are urgently needed. Regulators already require “may trigger shellfish allergy” statements and call for post-market surveillance; EFSA opinions on *T. molitor* and *A. domesticus* highlight remaining data gaps on allergens, contaminants, and the heavy metal uptake from feed substrates [200]. Parallel work must refine microbiological controls and quantify lipid oxidation limits for high-PUFA insect oils.

Processing science will determine the cost parity with plant proteins. Emerging unit operations—ultrasound-assisted extraction that doubles the soluble cricket protein yield and improves emulsification [275] or two step air/tribo-static classification that increases protein to ≈45% without water or solvents [276,277]—show promise but remain at the pilot scale. Integrated schemes that valorize the 5–15% chitin fraction as food-grade chitosan could unlock the full utilization of whole biomass. Scaling will hinge on dedicated dryers, oil presses, and continuous blancher–grinders sized for ton-per-hour larvae streams, plus automated climate and harvest control in vertical farms.

Environmental credentials are strong at the farm gate yet are undercharacterized post-processing. Prospective life-cycle assessments that extend to protein isolate or oil refinery stages reveal that the electricity mix and formulated feeds can swing global warming or acidification scores toward those of poultry. Scenario modeling around renewable energy and waste stream diets will pinpoint net-positive configurations [257]. Future LCAs should incorporate biodiversity and welfare metrics now absent from most studies.

Consumer research confirms that “invisibility” drives uptake: the willingness to try jumps when insects are milled into flour or isolated and embedded in familiar foods, though neophobia, perceived naturalness, and taste concerns persist [278]. Messaging that emphasizes protein quality and sustainability, along with repeated exposure through tastings or chef endorsements, is recommended as a lever while regulatory harmonization (standard definitions for food-grade insect meal and global residue limits) is in progress.

Long-term productivity gains will stem from advancements in genetics and biotechnology. Selective breeding roadmaps outline gains in growth, feed conversion, and nutrient profiles but lack coordinated programs or genomic tools compared with livestock [279]. Entomoculture—the cultivation of insect muscle or fat cells in bioreactors—could eventually deliver allergen-reduced, fully disguisable insect protein at a lower cost than mammalian cell cultures yet still faces hurdles in lineage stabilization, scaffold design, and medium optimization [280]. Coupling improved strains with circular agro-systems (e.g., feeding brewery or legume residues and returning frass as fertilizer) will complete the sustainability loop and strengthen the case for policy incentives.

Collectively, progress in allergen profiling, benign processing, whole-system LCA, consumer engagement, and genetic improvement will transform insects from a niche novelty into a safe, scalable, and accepted pillar of the sustainable protein supply.

## 8. Conclusions

Edible insects emerge from the literature not only as an alternative protein but also as a multidimensional resource that can nourish people, stabilize supply chains, and reduce agriculture’s environmental footprint simultaneously. Comparative compositional data demonstrate that selected species provide complete, highly digestible proteins; heart-healthy lipids; and bioavailable micronutrients in concentrations that rival conventional meats while also supplying chitinous dietary fiber—an otherwise rare attribute in animal-derived foods. Equally important, modern fractionation techniques reveal insect proteins to be surprisingly versatile techno-functional agents: when defatted, pH-shifted, or mildly hydrolyzed, they emulsify, foam, gel, and bind water at levels comparable to soy or egg ingredients, enabling a seamless integration into familiar matrices such as breads, pastas, meat analogs, and ready-to-drink beverages without compromising sensory quality.

From a systems perspective, life-cycle assessments consistently assign insects a fraction of the land use, water demand, and greenhouse gas emissions associated with livestock. This advantage expands when rearing substrates come from food industry side streams and when renewable energy powers climate control. At the same time, cross-reactive allergens, flavor unfamiliarity, and fragmented regulatory frameworks temper the pace of adoption. The evidence suggests that these hurdles are manageable: controlled enzymatic hydrolysis can reduce allergenicity, targeted defatting and fermentation can enhance flavor, and emerging international standards are starting to normalize trade. Collectively, the insights reviewed here indicate that with continued investment in gentle, energy-efficient processing; transparent labeling; and consumer education, insect-derived ingredients can evolve from a niche novelty to trusted pillars of resilient, low-impact food systems.

## Figures and Tables

**Table 1:** Regulatory status of edible insects in Europe and United States [30].

Scientific Name (Common Name)	EU Novel Food Status	Typical US Market Status
*Tenebrio molitor* (Coleoptera: Tenebrionidae) (yellow mealworm, larva)	Authorized—dried, frozen, paste, and UV-treated powder (implementing Regs 2021/882 and 2022/169)	Widely marketed as whole larva and powder; no FDA objection when produced under CGMP
*Acheta domesticus* (Orthoptera: Gryllidae) (house cricket)	Authorized—frozen, dried, and partially defatted powder (Regs 2022/188 and 2023/5) (EUR-Lex)	Principal species in US retail flours and bars
*Locusta migratoria* (Orthoptera: Acrididae)	Authorized—frozen, dried, ground (Reg 2021/1975)	Sold chiefly as whole-insect snack; emerging powders
*Alphitobius diaperinus* (Coleoptera: Tenebrionidae) (lesser mealworm)	Authorized—frozen, paste, dried, powder (Reg 2023/58)	Commercial powders and savory snacks
*Hermetia illucens* (Diptera: Stratiomyidae) (black soldier fly)	EFSA opinion under review; not yet authorized for food; already authorized for feed	Pilot human food products; self-GRAS dossiers in preparation
*Gryllodes sigillatus* (Orthoptera: Gryllidae) (banded cricket)	Application submitted; no decision (dossier NF 2021/2313)	Niche US start-ups; GRAS self-determination
*Zophobas morio* (Coleoptera: Tenebrionidae) (king-mealworm)	No EU file to date	Limited US online sales
*Oecophylla smaragdina* (Hymenoptera: Formicidae) (Asian weaver ant)	Unregulated; traditional food in S-E Asia only	Not marketed for food
*Lethocerus indicus* (Hemiptera: Belostomatidae) (giant water bug)	Unregulated; traditional Thai/Vietnamese delicacy	Not marketed for food
*Imbrasia ertli* (Lepidoptera: Saturniidae) (saturniid moth caterpillar)	Unregulated; regional African consumption	Not marketed in EU/US
*Prionoplus reticularis* (Coleoptera: Cerambycidae) (Huhu beetle)	Unregulated	Not marketed
*Odontotermes* spp. (Blattodea: Termitidae) (subterranean termites)	Unregulated	Not marketed

**Table 2:** Protein functionality and nutritional quality of representative edible insect ingredients.

Ingredient (Species and Preparation)	Protein Solubility	Protein Digestibility	Protein Quality	Experimental Conditions/Notes
*A. domesticus* (House cricket)—*Whole/defatted*	~96% at pH 11; drops to ~11–15% near pI.	79–93% in vitro total protein digestibility (depending on processing) [42].	PDCAAS ≈ 84% (0.84)—limiting amino acid: Leucine [43].	PDCAAS measured in rats (ref. pattern 6 mo–3 yr child). Digestible indispensable amino acids (DIAASs) for cricket protein up to 89% for adults. High digestibility relative to plant proteins.
*T. molitor* (Yellow mealworm)—*Whole/defatted*	~97% at pH 11 (isolate); ~15% at pH 4 (near pI).	91–99% in vitro digestibility of protein (high unless over-dried) [42].	PDCAAS ~76–86% (limiting SAA: Met+Cys) [44].	PDCAAS from rat assays; higher PDCAAS reported with mild processing: essential amino acids sufficiently high to meet requirements except slightly low in sulfur AAs.
*B. mori* (Silkworm pupae)—*Protein concentrate*	High protein solubility in extracts (e.g., water-soluble fraction).	~90% (est.)—silkworm proteins are highly digestible [45].	PDCAAS ~99–100%—*complete* amino acid profile (limiting AA effectively none; Leu at 99–100%) [46].	PDCAAS determined via rat assay; exceptionally high foaming stability noted, possibly due to hydrophobic amino acid content.
*Cirina forda* (Lepidoptera: Saturniidae) (*African caterpillar*)—*Whole flour*	~90% at pH 5.5; solubility improves at extreme pH (55% at pH 11).	~85–87% in vivo digestibility, but lower net protein utilization due to amino acid imbalance [47].	PDCAAS ~42%—very low. *Poor amino acid balance*—deficient in sulfur AAs [48].	Values from rat feeding tests. Low PDCAAS despite decent digestibility implies one or more essential AAs far below requirements.

**Table 3:** Summarizes the functional properties of selected insects.

Ingredient (Species and Form)	Water-Holding Capacity (WHC) (g/g)	Oil-Holding Capacity (OHC) (g/g)	Emulsifying Capacity (EC) (%)	Emulsion Stability (ES) (%)	Foaming Capacity (FC) (%)	Foam Stability (FS) (%)	Gelation (*w*/*v*)	Experimental Conditions/Notes
Mealworm larvae (*T. molitor*)—*Protein isolate* [123]	3.95 ± 0.2	2.74 ± 0.06	66.6 ± 2.2	51.3 ± 0.5	32.7 ± 0.9	30.3 ± 0.5	No gel	Solubility ~97% at pH 11; near pI (pH 4) solubility ~15%.
Locust (*Schistocerca gregaria* (Orthoptera: Acrididae) )—*Protein isolate* [123]	2.31 ± 0.19	3.22 ± 0.16	67.8 ± 1.6	50.4 ± 2.0	32.0 ± 1.9	6.2 ± 0.7	No gel	Solubility ~90% at pH 11. Low foam stability (only ~6%).
Cricket (G. sigillatus)—*Protein isolate* [124]	3.44 ± 0.13	3.33 ± 0.11	72.6 ± 1.9	62 ± 1.2	125 ± 25	92.0 ± 1.9	No gel	Extremely high foaming capacity and stability. Solubility 30% at pH = 3 ~96% and pH= 11.
Mealworm larvae (*T. molitor*)—*Whole flour* [125]	0.6 ± 0.19	0.71 ± 0.33	65.9 ± 1.5	27.6 ± 1.2	31.0 ± 1.4%	26.0 ± 0.9%	No gel	Non-defatted flour (~52% protein). Lower WHC/OHC than isolates; emulsions unstable (ES ~28%).
Locust (*S. gregaria*)—*Whole flour* [117,126]	2.17 ± 0.09	1.64 ± 0.06	69.2 ± 0.6	48.1 ± 0.6	22.3 ± 1.4	19.3 ± 0.9	No gel	High protein content (~76%) in flour. Emulsions quite stable (ES ~48%) even in whole flour form.
Cricket (*G. sigillatus*)—*Whole flour* [127]	2.34 ± 0.28	2.82 ± 0.08	62.0 ± 1.3	31.7 ± 0.9	41.0 ± 1.4	34.7 ± 2.8	No gel	Protein ~70% in flour. Balanced WHC/OHC ~2–3 g/g; moderate foam and emulsion stability.
African caterpillar (*C. forda*)—*Defatted flour* [126]	3.00 ± 0.00	3.58 ± 0.00	36.7 ± 0.1	45.4 ± 0.2	7.1 ± 0.2	3.0 ± 0.0	Gels at 6%	High solubility (~55%) achieved at pH 11. Shows exceptionally high water and oil binding (>>200%) after defatting. Minimum gelation concentration 6% (*w*/*v*).

**Table 4:** Bioactive functionality of peptide-rich ingredients derived from edible insects.

Ingredient (Preparation)	Cricket (*A. domesticus* Protein Hydrolysate) [152]	Silkworm (*B. mori* Pupae Protein Peptides) [153]	Locust (*S. gregaria* Protein Hydrolysate) [154]	Black Soldier Fly (*H. illucens*) [155]
Antioxidant Activity	2,2-diphenyl-1-picrylhydrazyl (DPPH) radical scavenging IC_50_ ≈ 455 µg/mL; 2,2′-Azino-bis(3-ethylbenzothiazoline-6-sulfonic acid) radical scavenging assay (ABTS) IC_50_ ≈ 71 µg/mL. Also 6.27 µmol TE/g (DPPH) and 19.5 µmol TE/g Ferric-Reducing Antioxidant Power (FRAP) in defatted-cricket Alcalase hydrolysate.	Moderate antioxidant capacity with hydrolysis yielding ~66% free-radical scavenging.	Relatively low antioxidant activity, peptides from *S. littoralis*, a related species, showed weak DPPH/FRAP activity.	DPPH: ~110 mmol TE/kg, FRAP: ~100 mmol TE/kg, ABTS: ~600 mmol TE/kg, depending on feed.
Antimicrobial Effects	Protein hydrolysates (PHs) show significant inhibition of collagenase and hyaluronidase, enzymes involved in skin aging and degradation of connective tissue [156]. An inhibitor from the gut contents of *A. domesticus* targets microsomal oxidation, particularly affecting Reduced Nicotinamide Adenine Dinucleotide Phosphate (NADPH) cytochrome c reductase, with greater activity against insect enzymes than mammalian ones.	Lebocin 1–3 (O-glycosylated, 51 aa) completely inhibit *Micrococcus luteus* at 3–6 µM; partial activity on *E. coli* (~25 µM) [157]. Gloverins (14 kDa glycine-rich) active vs. Gram-negative strains at 50–200 µM, isoform-dependent [158].	Orthopteran defensin-like peptides reported for locusts; typical MIC 0.1–5 µM vs. Gram-positive (*M. luteus*, *S. aureus*) and low-tens µM vs. Gram-negative bacteria. No direct data yet for protein hydrolysate, but presence of defensin genes suggests comparable potency.	Broad-spectrum antimicrobial: insect-derived chitosan (low MW) is effective against Gram(+) and Gram(−) bacteria. Minimum inhibitory concentrations are in the hundreds of µg/mL range (e.g., MIC > 500 µg/mL for *E. coli*).
Enzyme Inhibition	Protein hydrolysates (PHs) show significant inhibition of collagenase and hyaluronidase, enzymes involved in skin aging and degradation of connective tissue [156]. An inhibitor from the gut contents of *A. domesticus* targets microsomal oxidation, particularly affecting NADPH cytochrome c reductase, with greater activity against insect enzymes than mammalian ones [159].	Silkworm albumin fraction showed very potent ACE inhibition (IC_50_ = 0.047 mg/mL). Ultrasonication increases ACE-I activity ~40–67%.	Locust protein showed inhibition of pro-inflammatory enzymes (lipoxygenase and Cyclo-oxygenase (COX-2)) in vitro.	General literature suggests weak/no known ACE or Lipoxygenase (LOX) inhibition.
Immunomodulation [160]	Peptide-rich cricket meals up-regulate innate immune markers in vivo: in African catfish, inclusion of 10–30% cricket meal elevated lysozyme activity, total leukocyte count, and glutathione-based antioxidant enzymes, improving survival after Aeromonas challenge.	Polysaccharides (silkrose/dipterose) and chitin/chitosan fractions act as immunostimulants. Diets containing defatted silkworm pupae (25–50%). Serum activities of the antioxidant enzymes superoxide dismutase (SOD) and catalase (CAT) were significantly increased and reduced lipid peroxidation in mirror carp; White Blood Cell (WBC) counts rose in rainbow trout, and peptidoglycan recognition protein BmPGRP-S5-mediated phagocytosis toward *E. coli* was demonstrated.	Limited but emerging evidence: partial fishmeal replacement (≤25%) with Locusta meal maintains or improves hematological indices and glutathione S-transferase (GST) antioxidant activity in tilapia and catfish, indicating preserved non-specific immunity; high inclusion rates can depress these markers.	Multiple bioactive drivers—chitin, medium-chain fatty acids (lauric acid), and diet-dependent antimicrobial peptides (AMPs). Feeding trials show raised lysozyme, complement activity, leukocyte counts, and up-regulation of IL-1β, IL-17F, and TNF-α genes in crayfish, catfish, and sturgeon; in vitro digestion/fermentation of BSF meal releases high Short-Chain Fatty Acids (SCFAs) levels that can further modulate gut immunity.
Experimental Conditions/Notes	Antioxidant activity measured by DPPH and ABTS assays in vitro. Peptides obtained via enzymatic or gastrointestinal digestion. Defatting the cricket powder enhances peptide activity.	Silkworm protein hydrolysates produced by Alcalase, etc., exhibit strong angiotensin-converting enzyme (ACE)-inhibitory activity. Peptide Tyr-Ala-Asn from silkworm reduced blood pressure in hypertensive rats. Antioxidant assays (DPPH, etc.) indicate silkworm peptides can reach ~66% radical scavenging under optimal conditions.	Hydrolysate produced via simulated Gastro Intestinal digestion. The presence of peptides that inhibit COX-2/LOX suggests an anti-inflammatory potential beyond antioxidant activity (even if intrinsic antioxidant power is modest).	Measured using Trolox-Equivalent Antioxidant Capacity (TEAC)-DPPH, TEAC-FRAP, TEAC-ABTS, and Folin–Ciocalteu. In vitro digestion + fermentation simulated small/large intestine. Digestion phase accounted for >75% of activity. Fermentation led to high SCFA release in blood meal-fed larvae.

**Table 5:** Representative uses of edible insect ingredients in commercial-style food formulations. ↑—higher content; ↓—lower content.

Product Category and Format	Typical Insect Ingredient/Inclusion Level	Reported Benefits (Nutrition/Techno-Function/Sensory)	Representative Studies
Bakery (bread, biscuits, cookies, muffins, breakfast cereals, protein bars)	• Cricket or mealworm whole flour 5–15% (*w*/*w* dough) • Palm weevil larvae flour ≤70% in composite biscuits	↑ protein (+30–60%), improves EAA balance (adds Lys and Trp); ↑ Fe, Zn water-binding softens crumb; ≤10% keeps color and texture acceptable; flavor easily masked with cocoa/spices	[209,210,211]
Pasta and noodles	• Cricket/grasshopper flour 10–15% semolina • Mealworm flour 5–10%	↑ protein and iron; higher DIAASs; minor darkening but texture comparable to durum pasta; acceptance high when sauced	[212,213,214]
Meat products and extenders (sausages, patties, meatballs, burgers)	• Defatted cricket/locust/mealworm flour 5–15% of batter • Mealworm paste, 10% in hybrid burgers	Binds water and fat → lower cook loss; ↑ protein, Fe, Zn, PUFA; can lower SFA; texture/juiciness maintained; color slightly darker; allergen labeling needed	[181,215,216]
Meat analogs (high-moisture extrusion; jerky-style)	• Cricket flour + soy isolate (15–30% insect solids) • Insect/plant blends for jerky analogs	Forms fibrous “muscle-like” texture (anisotropic index ≤ 2.8); insect proteins supply complete AA profile; very high inclusion can lower tensile strength—blend optimization required	[181,217]
Extruded snacks and crisps	• Cricket/mealworm flour 5–20% with corn or rice grits	↑ protein 2–4 g per 30 g serving; expansion ratio ↓ above ~10% insect; crunch and flavor acceptable with seasoning	[218,219,220,221]
Dairy analogs and beverages (protein shakes, yogurt-type, kefir)	• Cricket/buffalo worm protein powder 5–12% (RTD shakes) • Silkworm pupae or buffalo worm flour replacing 5–10% milk solids	Whey-like protein boost; fermentation kinetics retained; ↑ Fe, Zn, vit B12; “nutty/earthy” flavor must be masked; slight beige color	[222,223]

## Data Availability

Data will be made available upon request.

## Abbreviations

The following abbreviations are used in this manuscript:

**Abbreviation**	**Meaning**
AA	amino acid
BSF	black soldier fly
CFU	colony-forming unit
CP	crude protein
DM	dry matter
EAA	essential amino acid
FAO	Food and Agriculture Organization of the United Nations
GHG	greenhouse gas
GRAS	generally recognized as safe
HPLC	high-performance liquid chromatography
IC_50_	half-maximal inhibitory concentration
LCA	life-cycle assessment
OHC	oil-holding capacity
PBM	population balance modeling
PUFA	polyunsaturated fatty acid
RSM	response surface methodology
SD	standard deviation
TEA	techno-economic analysis
WHC	water-holding capacity
